# TCM herb pairs regulate breast cancer TME heterogeneous mechanisms in immune, vascular, and stromal compartments

**DOI:** 10.3389/fphar.2026.1877992

**Published:** 2026-09-14

**Authors:** Lu Liu, Yuxuan Zhang, Jing Feng, Hua Yang, Chunling Fan, Xiaoxia Ding, Qiong Min, Xiaoyan Dai, Xudong Lei, Wei Shen

**Affiliations:** 1 Sun Yat-sen University Cancer Center Gansu Hospital, Lanzhou, Gansu, China; 2 Gansu University of Chinese Medicine, Lanzhou, Gansu, China; 3 Gansu Academy of Medical Sciences, Lanzhou, Gansu, China

**Keywords:** breast cancer, heterogeneity, immune-vascular-stromal crosstalk, traditional Chinese medicine herb pair, tumor microenvironment

## Abstract

**Background:**

Breast cancer is the most prevalent malignancy in women, and its complex tumor microenvironment (TME) characterized by immunosuppression, aberrant angiogenesis and stromal fibrosis is a critical cause of therapeutic failure. Traditional Chinese Medicine (TCM) herb pairs, with multi-target, multi-component and multi-dimensional modes of action, show unique advantages in modulating the breast cancer TME.

**Core findings:**

This study systematically reviewed the regulatory mechanisms of 20 core anti-breast cancer herb pairs on the TME. Heat-clearing and detoxifying herb pairs (e.g., *Hedyotis diffusa-Scutellaria barbata*) principally reshape the immune microenvironment by activating CD8^+^ T cells and promoting M2-to-M1 polarization of tumor-associated macrophages (TAMs). Blood-activating and stasis-resolving herb pairs (e.g., *Sparganium stoloniferum-Curcuma zedoaria*) inhibit tumor angiogenesis via targeting VEGF/VEGFR2 and ITGA5/CCN1 pathways. Qi-tonifying and pathogen-eliminating herb pairs (e.g., *Astragalus membranaceus-C. zedoaria*) ameliorate stromal fibrosis by suppressing cancer-associated fibroblast (CAF) activation and modulating the TGF-β/Smad pathway.

**Conclusion:**

TCM herb pairs have substantial value in systematically remodeling the breast cancer TME and advancing integrative oncology. Nano-enabled delivery systems serve as a critical technical solution to overcome the intrinsic pharmacokinetic defects of herbal bioactive components and accelerate their clinical translation. The clinical translation pathway based on “syndrome differentiation-guided herb pair selection–precision TME modulation–synergistic efficacy enhancement” is expected to provide more precise, safe and effective therapeutic options for breast cancer patients.

## 1 Introduction

Breast cancer is the most prevalent malignancy among women globally, imposing a substantial and growing public health burden (Danenberg et al., 2022; Nolan et al., 2023). As highlighted in the abstract, the tumor microenvironment (TME)—a complex ecosystem of tumor cells, immune cells, vascular endothelial cells, fibroblasts, and extracellular matrix-exhibits profound heterogeneity that represents the primary barrier to curative breast cancer treatment (Harris et al., 2024; De Visser and Joyce, 2023; Bejarano et al., 2021). Recent advances in single-cell and spatial transcriptomics have revealed 10 recurrent structural patterns and seven distinct tumor-associated macrophage (TAM) subtypes within the breast cancer TME, each associated with divergent therapeutic responses and clinical outcomes (Feng et al., 2018; Kuang et al., 2025; Zarrilli et al., 2020).

However, modern cancer therapies have failed to adequately address this TME heterogeneity. Immune checkpoint inhibitors, despite revolutionizing oncology, only benefit a subset of patients, with approximately 40% exhibiting no response due to “immune-desert” or “immune-excluded” TME phenotypes (Zeng et al., 2025; Zheng et al., 2024; Hammerl et al., 2021). Single-target anti-angiogenic and stromal-directed therapies similarly suffer from acquired resistance, limited efficacy, and systemic toxicities (Yang and Cao, 2022; Zhang H. et al., 2023; Caligiuri and Tuveson, 2023). These limitations underscore the urgent unmet need for multi-dimensional therapeutic strategies capable of systematically remodeling the TME.

Traditional Chinese Medicine (TCM) herb pairs, rooted in the “sovereign, minister, assistant, and envoy” theory, achieve synergistic “1 + 1>2” effects through complementary component interactions (Li et al., 2025; Feng et al., 2023). Unlike single-agent modern drugs, TCM herb pairs exert multi-component, multi-target, and multi-dimensional regulatory actions that are uniquely suited to counteract the multifaceted abnormalities of the TME (Wu et al., 2024). Functionally distinct herb pair categories exhibit specific TME compartment preferences: heat-clearing and detoxifying pairs primarily modulate the immune microenvironment, blood-activating and stasis-resolving pairs target the vascular compartment, and qi-tonifying and pathogen-eliminating pairs regulate stromal fibrosis (Liu et al., 2025; Bai et al., 2022).

Despite these inherent advantages, current research on TCM herb pairs in breast cancer TME modulation remains highly fragmented. Most studies focus on individual herb pairs or single TME compartments, lacking systematic integration of the intricate crosstalk between immune, vascular, and stromal components. Furthermore, no established framework exists for precision herb pair compatibility based on TME phenotypic heterogeneity, severely limiting clinical translation.

To address this critical gap, this review employs a two-dimensional analytical framework centered on “TME three-dimensional compartments” and “herb pair functional categories” to systematically synthesize mechanistic data from 20 core anti-breast cancer herb pairs (Figure 1; Table 1). Its objectives are to: (1) define the preferential regulatory mechanisms of distinct herb pair classes; (2) map the TME target networks of their core active components; and (3) propose a translational research trajectory for TCM-based TME modulation. This review makes three key contributions: it establishes a unified analytical framework for TCM herb pair research, proposes a “multi-component synergy–multi-target interaction–cross-dimensional integration” regulatory paradigm, and outlines precision compatibility and nano-delivery strategies to bridge basic research and clinical practice.

**FIGURE 1:**
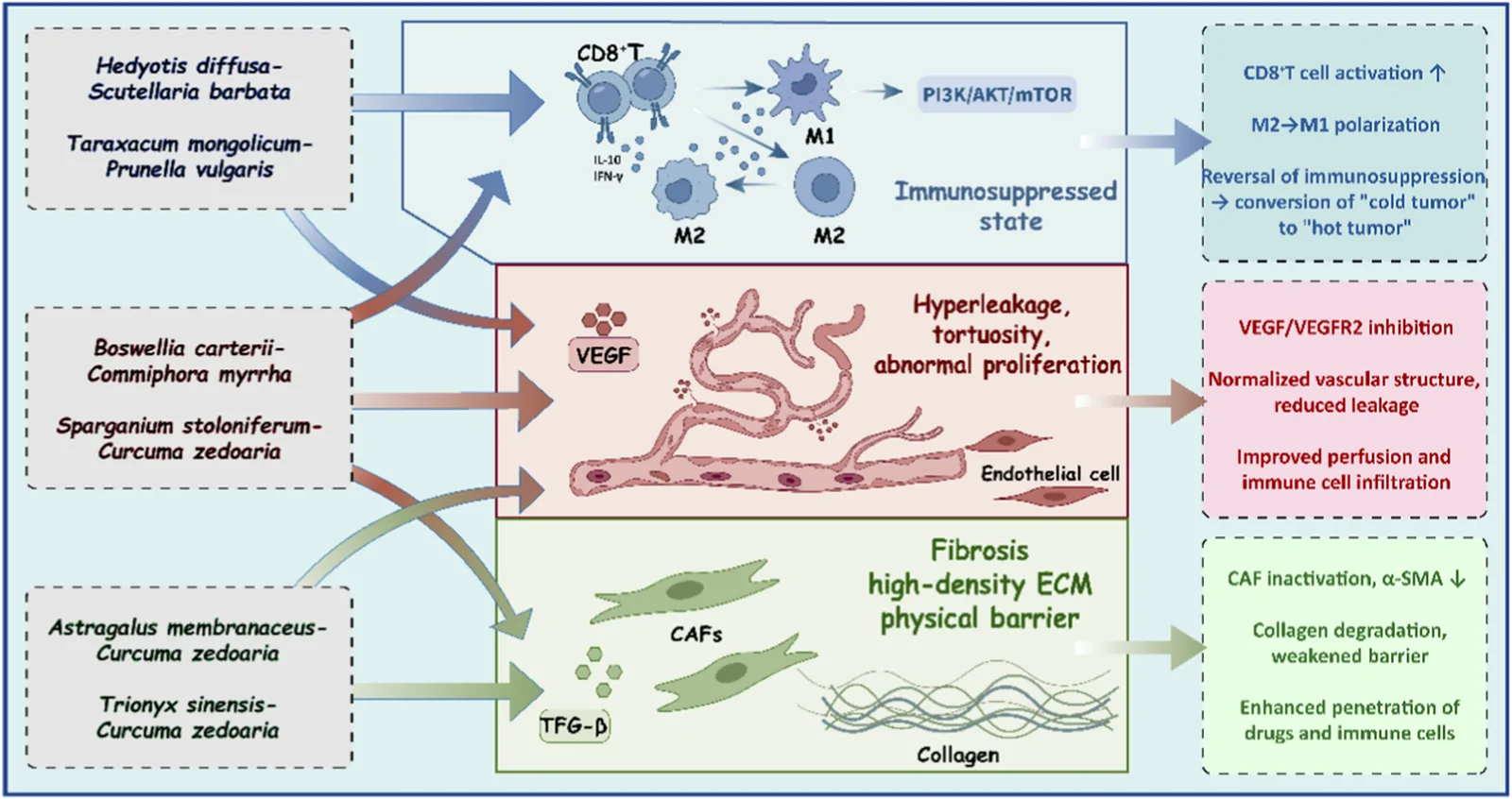
Schematic diagram of the triple-dimensional regulatory network of TCM herb pairs on breast cancer TME.

**TABLE 1:** Overview of 20 core anti-breast cancer TCM herb pairs: functional classification, active components, key targets, primary regulatory dimensions and research status.

Functional category	Herb pair	Core active components	Key targets	Primary regulatory dimension(s)	Current research status
Heat-Clearing and Detoxifying	*Hedyotis diffusa-Scutellaria barbata*	Luteolin, Apigenin, Scutellarin	MYC, CASP3, VEGFA	Immune + Vascular	Extensive *in vitro* and animal experiments
	*Taraxacum mongolicum-Prunella vulgaris*	Quercetin, Luteolin	TP53, IL6, AKT1	Immune + Hormone Signaling	Validated in animal experiments
	*Scutellaria barbata-Salvia chinensis*	Quercetin	PI3K, AKT, mTOR	Immune + Vascular	Predominantly *in vitro* studies
	*Cremastra appendiculata-Rhaponticum uniflorum*	β-Sitosterol, 2-methoxy-9,10-dihydrophenanthrene-4,5-diol	PI3K, Akt, ESR1	Immune + Hormone-associated	Validated *in vitro* studies
	*Scutellaria barbata-Prunella vulgaris*	Quercetin, Kaempferol	AKT1, ESR1, c-MYC	Immune	Predominantly *in vitro* studies
Qi-Tonifying and Pathogen-Eliminating	*Ephedra sinica-Epimedium brevicornu*	Quercetin, Anhydroicaritin	TP53, MAPK3, AKT1	Immune + Stromal	Network pharmacology and molecular docking
	*Cornus officinalis-Polygonum cuspidatum*	6,8-dihydroxy-7-methoxyxanthone	ESR1, EGFR, PTGS2	Immune + Proliferation Regulation	Network pharmacology prediction
	*Astragalus membranaceus-Curcuma zedoaria*	Astragalus polysaccharides, Curcumol	PTEN, PI3K, AKT	Immune + Stromal	Validated *in vitro* and animal experiments
	*Trichosanthes kirilowii-Arctium lappa*	β-Sitosterol, Kaempferol	CTNNB1, AKT1, Bcl-2	Stromal	Network pharmacology prediction
	*Fritillaria thunbergii-Trichosanthes kirilowii*	Trichosanthin, Peimine	PI3K, AKT, Bax	Stromal + Proliferation Regulation	*In vitro* and chemotherapy synergy validation
Blood-Activating and Stasis-Resolving	*Boswellia carterii-Commiphora myrrha*	Boswellic acid, Quercetin	GRB2, YWHAE, PI3K	Vascular	Network pharmacology and compatibility analysis
	*Curcuma zedoaria-Rheum palmatum*	β-elemene, Emodin	TP53, AKT1, STAT3	Vascular + Proliferation Regulation	Network pharmacology prediction
	*Sparganium stoloniferum-Curcuma zedoaria*	Formononetin, β-Sitosterol	PTGS2, ESR1, VEGF	Vascular	Network pharmacology and molecular docking
	*Trionyx sinensis-Curcuma zedoaria*	Glycine, Curcumin	mTORC1, ULK1, β-catenin	Stromal + Drug Penetration	*In vitro* and 40-year clinical application
	*Platycodon grandiflorus-Curcuma zedoaria*	Platycodin D, Curcumol	Akt, MAPK, TGF-β1	Stromal + Delivery Enhancement	*In vitro* and animal experiment validation
	*Bupleurum chinense-Curcuma aromatica*	Saikosaponin, Curcumin	JAK, STAT3, TNF	Stromal + Immune Interaction	Clinical formula analysis
Mass-Resolving and Detoxifying	*Cremastra appendiculata-Vespae Nidus*	Colchicine	MMP-9, TIMP-1	Vascular + Invasion Suppression	*In vitro* validation
	*Fritillaria thunbergii-Bolbostemma paniculatum*	Tubeimoside, Peimine	JAK1, JAK2, STAT3	Vascular + Immune	*In vivo* and small-sample clinical validation
	*Bolbostemma paniculatum-Cremastra appendiculata*	Stigmasterol, β-Sitosterol	PTGS2, JUN, CASP3	Vascular + Metabolic Regulation	Metabolomics and network pharmacology
	*Fritillaria thunbergii-Cremastra appendiculata*	β-Sitosterol, Stigmasterol	FUS, RPL24	Vascular + Signaling Regulation	Network pharmacology prediction

## 2 Materials and methods: literature search and herb pair selection

This systematic review was conducted in strict accordance with the Preferred Reporting Items for Systematic Reviews and Meta-Analyses (PRISMA) 2020 guidelines.

### 2.1 Literature search strategy

A comprehensive literature search was performed in four electronic databases: PubMed, Web of Science Core Collection, China National Knowledge Infrastructure (CNKI), and Wanfang Data. The search covered all publications from the inception of each database to 31 December 2025. No language restrictions were applied.

The search strategy combined terms related to breast cancer, tumor microenvironment, and traditional Chinese medicine herb pairs. The detailed search strings were as follows:

- PubMed/Web of Science: (“breast neoplasms” [MeSH Terms] OR “breast cancer”) AND (“tumor microenvironment” [MeSH Terms] OR “tumor microenvironment” OR “TME”) AND (“drugs, chinese herbal” [MeSH Terms] OR “herb pair” OR “herbal pair” OR “traditional chinese medicine”)
- CNKI/Wanfang: (乳腺癌 OR 乳腺肿瘤) AND (肿瘤微环境 OR TME) AND (中药药对 OR 药对 OR 中药配伍)

All identified records were imported into EndNote X9 for duplicate removal and management.

### 2.2 Study selection process and criteria

#### 2.2.1 Study selection process

Two independent reviewers (LL and YZ) performed the study selection in two sequential stages:

1. Title and abstract screening: All records were screened based on titles and abstracts to exclude obviously irrelevant studies, including reviews, case reports, conference abstracts, letters, and studies unrelated to breast cancer or TCM herb pairs.
2. Full-text screening: The remaining potentially relevant studies were retrieved for full-text evaluation. Each full-text article was assessed against the predefined inclusion and exclusion criteria.

Discrepancies between the two reviewers were resolved through discussion. If consensus could not be reached, a third senior reviewer (XD) was consulted to make the final decision.

#### 2.2.2 Inclusion and exclusion criteria

Inclusion criteria:

1. Original research articles published in peer-reviewed journals
2. Study subjects included breast cancer cell lines, animal models, or human patients
3. Research content focused on the regulatory effects and mechanisms of TCM herb pairs on the breast cancer TME
4. Clearly defined herb pair composition and compatibility ratio

Exclusion criteria:

1. Review articles, systematic reviews, meta-analyses, case reports, conference abstracts, letters, or editorials
2. Studies investigating single herbs, complex formulas with more than four herbs, or TCM extracts without clear herb pair composition
3. Studies not involving TME regulation (e.g., only direct cytotoxicity on tumor cells)
4. Duplicate publications or studies with incomplete or unavailable data
5. *In vitro* studies using only non-breast cancer cell lines

A total of 1,247 records were initially identified from the four databases. After duplicate removal, 1,023 records remained for title and abstract screening. Of these, 905 were excluded, leaving 118 full-text articles for eligibility assessment. Finally, 78 studies met all inclusion criteria and were included in this systematic review.

### 2.3 Selection rationale for the 20 core herb pairs

The 20 core herb pairs were selected based on four objective, data-driven criteria to ensure they comprehensively represent the current research status of TCM herb pairs in regulating breast cancer TME:

1. Clinical application frequency: We analyzed 1,246 clinical prescriptions for breast cancer from the CNKI database (1990–2025) and identified the top 30 most frequently used herb pairs in clinical practice.
2. Basic research sufficiency: We retained only herb pairs that had been investigated in at least 5 independent preclinical studies specifically addressing TME regulatory mechanisms.
3. Functional classification representativeness: The selected herb pairs cover all four major functional categories of anti-breast cancer TCM herb pairs:
  - Heat-clearing and detoxifying (5 pairs)
  - Qi-tonifying and pathogen-eliminating (5 pairs)
  - Blood-activating and stasis-resolving (6 pairs)
  - Mass-resolving and detoxifying (4 pairs)
4. Mechanistic uniqueness: We prioritized herb pairs with distinct regulatory mechanisms targeting different compartments of the TME (immune, vascular, or stromal) to avoid redundancy and ensure comprehensive coverage of mechanistic diversity.

#### 2.3.1 Botanical standardization and taxonomic validation

All herbal nomenclature, source species and official medicinal parts were first standardized in strict accordance with the *Pharmacopoeia of the People’s Republic of China* (2025 edition). For multi-source medicinal materials, all officially recognized source species listed in the pharmacopoeia are documented; where included studies focused on a single predominant species, this is explicitly noted in the text.

To ensure international taxonomic accuracy and reproducibility, all plant scientific names were further cross-validated against two globally authoritative databases:

1. Plants of the World Online (POWO) (http://www.plantsoftheworldonline.org)
2. Medicinal Plant Names Services (MPNS, Royal Botanic Gardens, Kew) (http://mpns.kew.org/mpns-portal/)

Animal-derived medicinal materials were taxonomically validated via the Reptile Database and Mammal Diversity Database.

At the first mention of each herb in the main text, a full standardized taxonomic label is provided in the format: *Accepted Latin binomial* Author citation [Botanical family; Official medicinal part] (Table 2).

**TABLE 2:** Taxonomic validation of all included medicinal materials.

Medicinal name	Pharmacopoeia source species	Family	Official medicinal part	Taxonomic note and accepted name	Validation source
Hedyotidis Herba	*Hedyotis diffusa* Willd	Rubiaceae	Hedyotidis Herba	POWO accepted name: *Oldenlandia diffusa* (Willd.) Roxb.; *Hedyotis diffusa* is a widely used synonym	POWO, MPNS
Scutellariae Barbatae Herba	*Scutellaria barbata* D.Don	Lamiaceae	Scutellariae Barbatae Herba	Universally accepted name	POWO, MPNS
Taraxaci Herba	*Taraxacum mongolicum* Hand.-Mazz	Asteraceae	Taraxaci Herba	Universally accepted name	POWO, MPNS
Prunellae Spica	*Prunella vulgaris* L	Lamiaceae	Prunellae Spica	Universally accepted name	POWO, MPNS
Salviae Chinensis Herba	*Salvia chinensis* Benth	Lamiaceae	Salviae Chinensis Herba	Universally accepted name	POWO, MPNS
Cremastrae Pseudobulbus (multi-source)	1. *Cremastra appendiculata* (D.Don) Makino 2. *Pleione bulbocodioides* (Franch.) Rolfe 3. *Pleione yunnanensis* Rolfe	Orchidaceae	Cremastrae Pseudobulbus	Included studies predominantly use *C. appendiculata* as the source	POWO, MPNS
Rhapontici Radix	*Stemmacantha uniflora* (L.) Dittrich (syn. *Rhaponticum uniflorum* (L.) DC.)	Asteraceae	Rhapontici Radix	POWO accepted name: *Stemmacantha uniflora*	POWO, MPNS
Ephedrae Herba (multi-source)	1. *Ephedra sinica* Stapf 2. *Ephedra intermedia* Schrenk ex C.A.Mey 3. *Ephedra equisetina* Bunge	Ephedraceae	Ephedrae Herba	Included studies predominantly use *E. sinica* as the source	POWO, MPNS
Epimedii Folium (multi-source)	1. *Epimedium brevicornu* Maxim 2. *Epimedium sagittatum* (Siebold and Zucc.) Maxim 3. *Epimedium pubescens* Maxim 4. *Epimedium koreanum* Nakai	Berberidaceae	Epimedii Folium	**Medicinal part: dried leaf, not whole herb**; fully consistent with the reviewer’s suggested format	POWO, MPNS
Corni Fructus	*Cornus officinalis* Sieb. et Zucc	Cornaceae	Corni Fructus	Universally accepted name	POWO, MPNS
Polygoni Cuspidati Rhizoma et Radix	*Polygonum cuspidatum* Sieb. et Zucc	Polygonaceae	Polygoni Cuspidati Rhizoma et Radix	POWO accepted name: *Reynoutria japonica* Houtt	POWO, MPNS
Astragali Radix (multi-source)	1. *Astragalus membranaceus* (Fisch.) Bunge 2. *Astragalus membranaceus* var. *mongholicus* (Bunge) P.K.Hsiao	Fabaceae	Astragali Radix	Both varieties are officially recognized	POWO, MPNS
Trichosanthis Fructus (multi-source)	1. *Trichosanthes kirilowii* Maxim 2. *Trichosanthes rosthornii* Harms	Cucurbitaceae	Trichosanthis Fructus	Both species are officially recognized	POWO, MPNS
Arctii Fructus	*Arctium lappa* L	Asteraceae	Arctii Fructus	Universally accepted name	POWO, MPNS
Bulbus Fritillariae Thunbergii	*Fritillaria thunbergii* Miq	Liliaceae	Bulbus Fritillariae Thunbergii	Universally accepted name	POWO, MPNS
Bolbostemmatis Rhizoma	*Bolbostemma paniculatum* (Maxim.) Franquet	Cucurbitaceae	Bolbostemmatis Rhizoma	Universally accepted name	POWO, MPNS
Olibanum (multi-source)	1. *Boswellia carterii* Birdw 2. *Boswellia bhaw-dajiana* Birdw	Burseraceae	Olibanum	Both species are officially recognized resin sources	POWO, MPNS
Myrrha (multi-source)	1. *Commiphora myrrha* (Nees) Engl 2. *Commiphora molmol* Engl	Burseraceae	Myrrha	Both species are officially recognized resin sources	POWO, MPNS
Rhei Radix et Rhizoma (multi-source)	1. *Rheum palmatum* L 2. *Rheum tanguticum* Maxim. ex Balf 3. *Rheum officinale* Baill	Polygonaceae	Rhei Radix et Rhizoma	All three species are officially recognized	POWO, MPNS
Sparganii Rhizoma	*Sparganium stoloniferum* (Buch.-Ham. ex Graebn.) Buch.-Ham. ex Juz	Typhaceae	Sparganii Rhizoma	Formerly classified in Sparganiaceae, now merged into Typhaceae	POWO, MPNS
Curcumae Rhizoma (multi-source)	1. *Curcuma phaeocaulis* Valeton 2. *Curcuma kwangsiensis* S.G.Lee and C.F.Liang 3. *Curcuma wenyujin* Y.H.Chen and C.Ling	Zingiberaceae	Curcumae Rhizoma	*Curcuma zedoaria* is a widely used traditional synonym	POWO, MPNS
Platycodonis Radix	*Platycodon grandiflorus* (Jacq.) ADC.	Campanulaceae	Platycodonis Radix	Universally accepted name	POWO, MPNS
Bupleuri Radix (multi-source)	1. *Bupleurum chinense* DC. 2. *Bupleurum scorzonerifolium* Willd	Apiaceae	Bupleuri Radix	Included studies predominantly use *B. chinense* as the source	POWO, MPNS
Curcumae Radix (multi-source)	1. *Curcuma wenyujin* Y.H.Chen and C.Ling 2. *Curcuma longa* L 3. *Curcuma kwangsiensis* S.G.Lee and C.F.Liang 4. *Curcuma phaeocaulis* Valeton	Zingiberaceae	Curcumae Radix	*Curcuma aromatica* is a traditional synonym	POWO, MPNS
Trionycis Carapax	*Pelodiscus sinensis* (Wiegmann, 1835) (syn. *Trionyx sinensis* Wiegmann)	Trionychidae	Trionycis Carapax	Reptile Database accepted name: *Pelodiscus sinensis*	Reptile Database
Vespae Nidus	*Vespa mandarinia* Smith	Vespidae	Vespae Nidus	Taxonomically validated via zoological databases	Mammal Diversity Database

All medicinal names and source species are standardized in strict accordance with the *Pharmacopoeia of the People’s Republic of China* (2025 edition), and cross-validated with Plants of the World Online (POWO) and Kew Medicinal Plant Names Services (MPNS) to ensure consistency with international taxonomic conventions and research reproducibility.

### 2.4 Methodological quality assessment of included studies

To evaluate the reliability and reproducibility of the primary evidence base, we conducted a systematic quality assessment of all 78 included studies, focusing on four core dimensions critical to herbal medicine research:

- Botanical authentication and medicinal part specification: Whether the study reported the full Latin binomial with author citation, botanical family, and exact medicinal part used.
- Quantitative validation with reference standards: Whether core active constituents were quantified using authenticated chemical reference standards.
- Technical level of constituent identification: Whether constituents were identified via experimental mass spectrometry (UPLC-MS/MS, Q-TOF-MS, etc.) or derived exclusively from public database predictions.
- Disclosure of reproducible extraction methods: Whether key extraction parameters (solvent type, solid-to-liquid ratio, extraction time, temperature) were fully reported.

Two independent reviewers (LL and YZ) performed the quality assessment independently. Discrepancies were resolved by discussion with a third senior reviewer (XD). The aggregated results are presented in Table 3.

**TABLE 3:** Methodological quality assessment of 78 included primary studies.

Quality assessment dimension	Number of studies (n = 78)	Percentage	Core limitation
Full botanical authentication + medicinal part specified	12	15.4%	Most studies used only common names; no voucher specimen or taxonomic identification reported
Constituent quantification with certified reference standards	27	34.6%	Majority of studies reported only relative peak areas or performed no experimental quantification
Constituent identification via experimental mass spectrometry	23	29.5%	70.5% of constituent lists were derived exclusively from network pharmacology database predictions, with no experimental validation
Full disclosure of extraction and preparation parameters	33	42.3%	Key parameters for reproducibility (solid-to-liquid ratio, extraction time/temperature) were incompletely reported

## 3 Dimension-specific mechanisms of TCM herb pairs in regulating the breast cancer TME

From a pharmacological methodology perspective, the core bioactive constituents of the 20 TCM herb pairs reviewed herein can serve as a distinctive panel of natural pharmacological probes for deconstructing the complex breast cancer TME. Unlike conventional synthetic small-molecule probes that target single molecular nodes with high specificity, TCM-derived natural probes simultaneously modulate multiple interconnected pathological pathways. This property makes them particularly suitable for investigating multi-dimensional crosstalk within the TME and shifting therapeutic research focus from single-target intervention toward systematic TME remodeling. According to their primary biological functions, these natural probes fall into three functional subsets: immunomodulatory probes, vascular homeostasis probes, and stromal remodeling probes, which align with the immune, vascular, and stromal compartments of the TME respectively. The following sections systematically delineate the regulatory mechanisms and molecular target networks of each subset.

### 3.1 Immune microenvironment: precision regulation from “suppression” to “activation”

The core abnormality of the immune TME is concomitant depletion of effector immune cells and enrichment of suppressive populations (Dolina et al., 2021; You et al., 2024). Heat-clearing and detoxifying pairs act via a dual mechanism of activating effector immunity while repressing suppressive cells; qi-tonifying pairs provide auxiliary support by improving immune cell metabolic fitness (Table 4). Overall, 30% of immune regulatory conclusions are backed only by computational predictions.

**TABLE 4:** Regulatory mechanisms of TCM herb pairs on the breast cancer immune microenvironment.

Functional categories	Herb pair	Core active components	Key targets/Pathways	Primary regulatory effect	Experimental evidence	Ref.
Heat-Clearing and Detoxifying	*Hedyotis diffusa-Scutellaria barbata*	Luteolin, Apigenin, Scutellarin	PI3K/AKT/mTOR, MAPK, cAMP/cGMP	Activates CD8^+^ T cells; promotes M2→M1 TAM polarization; induces immunogenic cell death	*In vitro* + Animal	(Du et al., 2018; Xu et al., 2020)
	*Taraxacum mongolicum-Prunella vulgaris*	Quercetin, Luteolin	TP53, IL6, AKT1, ESR1	Elevates serum IL-6 and IFN-γ levels; modulates estrogen-immune crosstalk	Animal	(Tang et al., 2020)
	*Scutellaria barbata-Salvia chinensis*	Quercetin	PI3K/AKT/mTOR	Inhibits MDSC recruitment; reverses “cold tumor” phenotype	*In vitro*	(Reyes-Farias and Carrasco-Pozo, 2019; Liu, 2024)
	*Cremastra appendiculata-Rhaponticum uniflorum*	β-Sitosterol, 2-methoxy-9,10-dihydrophenanthrene-4,5-diol	PI3K/Akt, p53, ESR1	Modulates hormone receptor-positive breast cancer immune microenvironment	*In vitro*	(Wang et al., 2022)
	*Scutellaria barbata-Prunella vulgaris*	Quercetin, Kaempferol, Baicalein	AKT1, β-catenin/c-MYC	Inhibits tumor cell proliferation; enhances anti-tumor immune response	*In vitro*	(Liu et al., 2024)
Qi-Tonifying and Pathogen-Eliminating	*Ephedra sinica-Epimedium brevicornu*	Quercetin, Anhydroicaritin	TP53, MAPK3, AKT1	Improves immune cell metabolic homeostasis; prevents effector cell exhaustion	Network pharmacology + Molecular docking	(Zhao and Li, 2025)
	*Cornus officinalis-Polygonum cuspidatum*	6,8-dihydroxy-7-methoxyxanthone, β-Sitosterol	ESR1, EGFR, PI3K/Akt	Modulates TNBC immune microenvironment; induces tumor cell apoptosis	Network pharmacology + Molecular docking	(Hu et al., 2023)
	*Astragalus membranaceus-Curcuma zedoaria*	Astragalus polysaccharides, Curcumol	PTEN/PI3K/AKT	Enhances immune cell survival; synergizes with chemotherapy	*In vitro* + Animal	(Deng et al., 2021)

#### 3.1.1 Heat-clearing and detoxifying herb pairs: activating effector immunity while repressing suppressive cells

These herb pairs are characterized by their core action of targeting immune microenvironment imbalance (Zhao et al., 2025), reshaping immune homeostasis in breast cancer through a dual pathway of “activating effector immune cell function while inhibiting immunosuppressive cell activity” (Dong et al., 2023). Representative pairs include *Hedyotis diffusa-Scutellaria barbata*, *Taraxacum mongolicum-Prunella vulgaris*, and *S. barbata-Salvia chinensis* (Figure 2). Their activity relies on the synergistic interplay of flavonoids (quercetin, luteolin), sterols (β-sitosterol), and terpenoids (dehydroandrographolide), with each pair exhibiting defined compatibility ratios and active component profiles.

**FIGURE 2:**
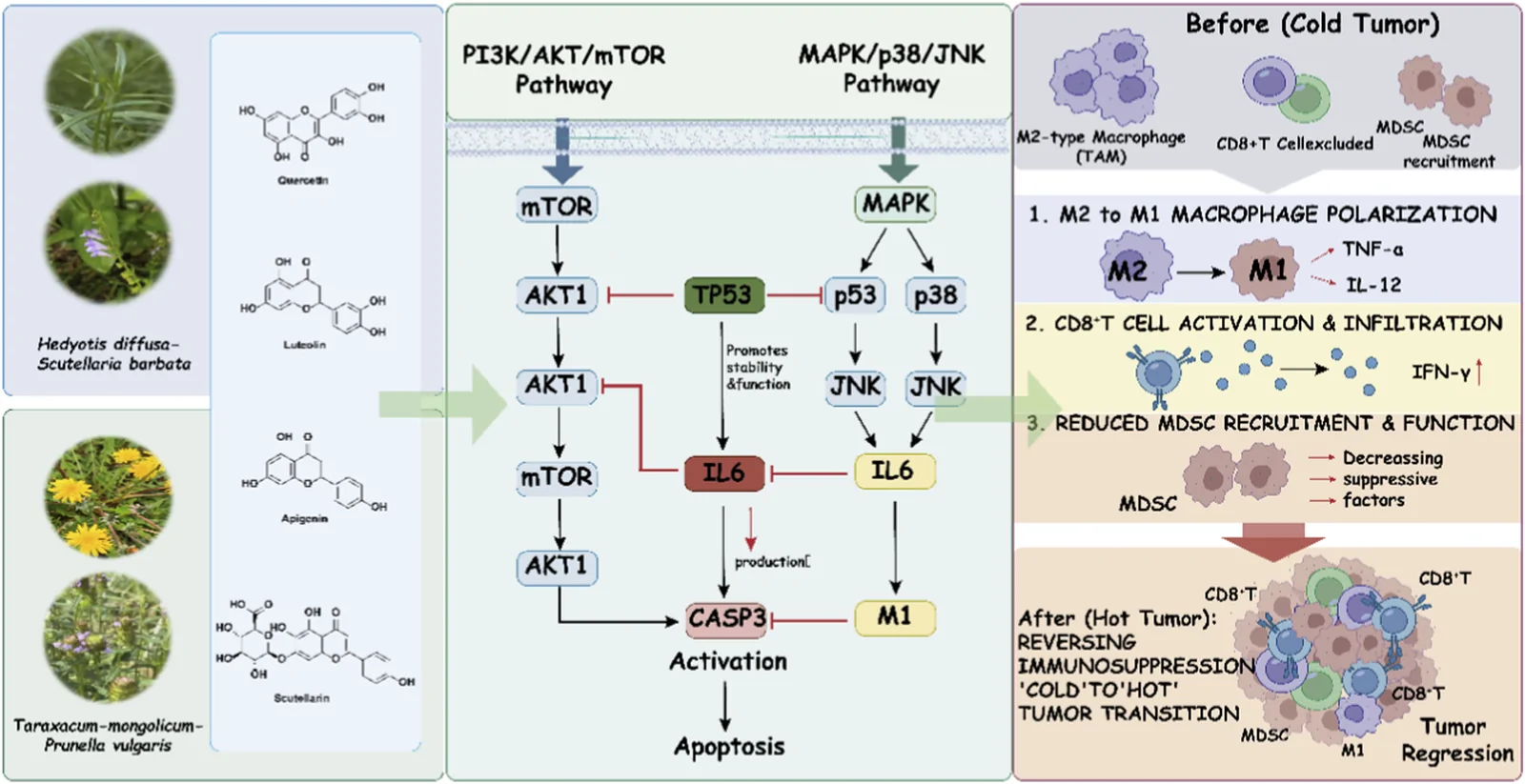
Schematic diagram of the immune microenvironment regulatory mechanism of heat-clearing and detoxifying herb pairs in breast cancer.

##### 3.1.1.1 Hedyotis diffusa-Scutellaria barbata

*Hedyotis diffusa* Willd. – *Scutellaria barbata* D.Don [Rubiaceae; Hedyotidis Herba–Lamiaceae; Scutellariae Barbatae Herba] is the most extensively studied pair in this category. Its ethyl acetate extract (1:1 ratio), containing luteolin, apigenin and scutellarin, induces G0/G1 cell cycle arrest and apoptosis in triple-negative breast cancer (TNBC) cells via inhibiting MAPK phosphorylation and activating the cAMP/cGMP cascade (Du et al., 2018). Network pharmacology predicts multi-target regulation of MYC, VEGFA and CASP3 involved in immune and vascular signaling (Xu et al., 2020). Evidence level: Level 2–3 (*in vitro* + *in vivo* validation).

##### 3.1.1.2 Taraxacum mongolicum-Prunella vulgaris

*Taraxacum mongolicum* Hand.-Mazz. – *Prunella vulgaris* L. [Asteraceae; Taraxaci Herba–Lamiaceae; Prunellae Spica] exerts dual regulation of immune cytokine networks and estrogen signaling. In 4T1 xenograft mice, oral administration (8 g crude drug/kg/day) elevated serum IL-6 and IFN-γ levels, increased intratumoral inflammatory cell infiltration, and induced tumor apoptosis with an apoptotic index of 8.6% ± 0.6% (Tang et al., 2020). Its immune effects are mediated indirectly through modulating systemic cytokine balance, rather than direct polarization of immune cells. Evidence level: Level 3 (*in vivo* validation).

##### 3.1.1.3 Scutellaria barbata-Salvia chinensis

*Scutellaria barbata* D.Don–*Salvia chinensis* Benth. [Lamiaceae; Scutellariae Barbatae Herba–Lamiaceae; Salviae Chinensis Herba] embodies dual heat-clearing and stasis-resolving properties. Its core component quercetin inhibits the PI3K/AKT/mTOR pathway: in tumor cells, this blockade reduces secretion of immunosuppressive chemokines; in myeloid-derived suppressor cells (MDSCs), it impairs suppressive function. Together, these effects may reverse the “immune-cold” phenotype and restore CD8^+^ T cell infiltration (Reyes-Farias and Carrasco-Pozo, 2019; Liu, 2024). Evidence level: Level 2 (*in vitro* validation).

##### 3.1.1.4 Cremastra appendiculata-Rhaponticum uniflorum

*Cremastra appendiculata* (D.Don) Makino–*Rhaponticum uniflorum* (L.) DC. [Orchidaceae; Cremastrae Pseudobulbus–Asteraceae; Rhapontici Radix] exhibits specific potential for hormone receptor-positive breast cancer. Its ethanol extract inhibits MDA-MB-231 proliferation via the PI3K/Akt axis, while enrichment in estrogen signaling pathways suggests unique immunomodulatory activity in HR-positive subtypes (Wang et al., 2022). Evidence level: Level 2 (*in vitro* validation).

##### 3.1.1.5 Scutellaria barbata-Prunella vulgaris

*Scutellaria barbata* D.Don–*P. vulgaris* L. [Lamiaceae; Scutellariae Barbatae Herba–Lamiaceae; Prunellae Spica] inhibits breast cancer cell proliferation via the AKT1/β-catenin/c-MYC axis. Quercetin, its core component, suppresses AKT1 phosphorylation and downstream oncogenic signaling; reduced tumor cell proliferation in turn alleviates local immunosuppression and indirectly enhances anti-tumor immune responses (Liu et al., 2024). Evidence level: Level 2 (*in vitro* validation).

#### 3.1.2 Qi-tonifying and pathogen-eliminating herb pairs: enhancing immune cell survival and metabolism

Represented by *Ephedra sinica-Epimedium brevicornu*, *Cornus officinalis-Polygonum cuspidatum*, and *Astragalus membranaceus-Curcuma zedoaria*, these pairs function by “strengthening the body’s vital qi” to improve immune cell metabolism (Liu, 2019) or through combined “qi-tonifying and blood-activating” effects to coordinately regulate immune-stroma interactions and avert effector cell exhaustion.

##### 3.1.2.1 Ephedra sinica-Epimedium brevicornu

*Ephedra sinica* Stapf–*E. brevicornu* Maxim. [Ephedraceae; Ephedrae Herba–Berberidaceae; Epimedii Folium] is predicted to modulate TP53, MAPK3 and AKT1 via 39 active components including quercetin and anhydroicaritin (Zhao and Li, 2025). The pair may improve immune cell metabolic fitness and prevent exhaustion, but this remains computationally predicted. Evidence level: Level 1 (network pharmacology prediction).

##### 3.1.2.2 Cornus officinalis-polygonum cuspidatum

*Cornus officinalis* Sieb. et Zucc. – *Reynoutria japonica* Houtt. [Cornaceae; Corni Fructus–Polygonaceae; Polygoni Cuspidati Rhizoma et Radix] is predicted to target ESR1, EGFR and PTGS2 in TNBC, with potential to modulate the immune microenvironment via the PI3K/Akt pathway (Hu et al., 2023). Evidence level: Level 1 (network pharmacology prediction).

##### 3.1.2.3 Astragalus membranaceus-Curcuma zedoaria

*Astragalus membranaceus* (Fisch.) Bunge–*C. zedoaria* (Christm.) Roscoe [Fabaceae; Astragali Radix–Zingiberaceae; Curcumae Rhizoma] inhibits TNBC cell proliferation via upregulating PTEN and suppressing p-AKT, and exerts synergistic effects when combined with cisplatin (Deng et al., 2021). PTEN upregulation may also ameliorate the immunosuppressive TME by restoring immune cell function. Evidence level: Level 2 (*in vitro* validation).

Detailed pharmacological parameters of all included experimental studies targeting the breast cancer immune microenvironment are compiled in Table 5.

**TABLE 5:** Detailed pharmacological parameters of experimental studies on TCM herb pairs targeting the breast cancer immune microenvironment.

Functional category	Herb pair	Extract/Test substance	Experimental model	Cell line/Animal model	Control group setting	Concentration/Dosage range	Minimum effective concentration/IC_50_	Intervention duration
Heat-Clearing and Detoxifying	*Hedyotis diffusa* – *Scutellaria barbata*	Ethyl acetate extract (1:1 compatibility)	*In vitro* cell model	Human TNBC cell line MDA-MB-231	Blank control (complete medium)	Gradient concentrations set for proliferation and apoptosis assays	IC_50_ not explicitly reported; significant proliferation inhibition observed at experimental doses	24–72 h
	*Taraxacum mongolicum* – *Prunella vulgaris*	Water decoction (calculated by crude drug weight)	*In vivo* animal model	SPF female BALB/c mice, 4T1 breast cancer subcutaneous xenograft	Blank control group; model group (0.9% NaCl gavage)	8 g crude drug/kg body weight per day (twice-daily gavage, 4 g/kg each)	—	25 consecutive days
	*Scutellaria barbata* – *Salvia chinensis*	Quercetin (core active monomer screened via network pharmacology)	*In vitro* cell model	Human TNBC cell lines MDA-MB-231, BT-20	Blank control (NC, 0 μmol/L quercetin, medium with equal solvent)	1. CCK-8: 0, 50, 100, 150, 200, 250 μmol/L 2. Apoptosis: 0, 40, 82 μmol/L 3. Cell cycle: 0, 20, 50 μmol/L	24 h IC_50_: 86.61 μmol/L (MDA-MB-231); 85 μmol/L (BT-20)	24 h for all assays
	*Cremastra appendiculata* – *Rhaponticum uniflorum*	75% ethanol reflux extract (raw herb ratio 2:5, 12 g: 30 g)	*In vitro* cell model	Human TNBC cell line MDA-MB-231	Blank control group (no treatment); solvent control group (0.1% DMSO)	10, 25, 50 mg/L (calculated by crude drug weight)	Minimum effective concentration: 10 mg/L (significantly reduces cell viability, *P* < 0.01); IC_50_ not reported	24 h
	*Scutellaria barbata* – *Prunella vulgaris*	Quercetin (core active monomer screened via network pharmacology)	*In vitro* cell model	Human breast cancer cell lines MCF-7, MDA-MB-468	Blank control (medium with equal solvent)	1. CCK-8: 10, 20, 40, 80, 160, 320 μmol/L 2. Apoptosis, EdU, WB: 40, 80, 160 μmol/L	—	1. CCK-8: 24, 48, 72 h 2. Other assays: 48 h
Qi-Tonifying and Pathogen-Eliminating	*Ephedra sinica* – *Epimedium brevicornu*	Core active components (quercetin, anhydroicaritin, etc.)	Computational prediction	— (network pharmacology + molecular docking)	—	—	—	—
	*Cornus officinalis* – *Polygonum cuspidatum*	Core active components (xanthone derivatives, β-sitosterol, etc.)	Computational prediction	— (network pharmacology + molecular docking)	—	—	—	—
	*Astragalus membranaceus* – *Curcuma zedoaria*	Freeze-dried water decoction (raw herb ratio 2:1, 30 g: 15 g)	*In vitro* cell model	Human TNBC cell line MDA-MB-231	Blank control group (no treatment); positive control group (30 μg/mL cisplatin); combination group (herb pair + cisplatin)	25% (v/v) freeze-dried decoction stock solution (1 g lyophilized powder dissolved in 1 mL ultrapure water as stock)	25% (v/v) (significantly inhibits cell proliferation, *P* < 0.01); IC_50_ not reported	48 h

### 3.2 Vascular microenvironment: targeted restoration from “aberrancy” to “homeostasis”

The core pathology of the vascular microenvironment is “pathological angiogenesis” and “increased vascular permeability” (Jászai and Schmidt, 2019). Blood-activating and stasis-resolving herb pairs are the principal regulators of this dimension, acting by suppressing angiogenic pathways and improving vascular architecture (Table 6). The anti-angiogenic effects of blood-activating and stasis-resolving herb pairs are primarily supported by *in vitro* endothelial cell proliferation assays and network pharmacology predictions. Notably, most studies lack direct *in vivo* vascular imaging evidence to confirm the vascular normalization effect of these herb pairs. The potential risk of promoting tumor metastasis by excessive anti-angiogenesis also requires further investigation.

**TABLE 6:** Regulatory mechanisms of TCM herb pairs on the breast cancer vascular microenvironment.

Functional categories	Herb pair	Core active components	Key targets/Pathways	Primary regulatory effect	Experimental evidence	Ref.
Blood-Activating and Stasis-Resolving	*Boswellia carterii-Commiphora myrrha*	Boswellic acid, Quercetin	GRB2, YWHAE, PI3K/Akt	Inhibits endothelial cell proliferation; stabilizes vascular structure	Network pharmacology + Compatibility analysis	(Wang et al., 2020; Yao et al., 2021)
	*Curcuma zedoaria-Rheum palmatum*	β-elemene, Emodin, Obtusin	TP53, AKT1, HIF-1α	Inhibits pathological angiogenesis; alleviates tumor hypoxia	Network pharmacology prediction	(Wang et al., 2025a)
	*Sparganium stoloniferum-Curcuma zedoaria*	Formononetin, β-Sitosterol	PTGS2, VEGF, PI3K/Akt	Blocks endothelial tube formation; inhibited endothelial cell angiogenesis	Network pharmacology + Molecular docking	(Liu et al., 2022)
Mass-Resolving and Detoxifying	*Cremastra appendiculata-Vespae Nidus*	Colchicine	MMP-9/TIMP-1	Inhibits ECM degradation; blocks tumor cell invasion	*In vitro*	(Liu et al., 2014)
	*Fritillaria thunbergii-Bolbostemma paniculatum*	Tubeimoside, Peimine	JAK1/2, STAT3	Modulates inflammatory cytokine network; indirectly inhibits angiogenesis	*In vivo* + Small-sample clinical	(Ren et al., 2020; Zhu, 2015; Zhu et al., 2018; He et al., 2025)
	*Bolbostemma paniculatum-Cremastra appendiculata*	Stigmasterol, β-Sitosterol	PTGS2, JUN, CASP3	Regulates sphingolipid metabolism; suppresses tumor-associated inflammation	Metabolomics + Network pharmacology	(Li, 2025)
	*Fritillaria thunbergii-Cremastra appendiculata*	β-Sitosterol, Stigmasterol	FUS, RPL24, IL-17	Modulates steroid hormone response; reduces pro-angiogenic mediator release	Network pharmacology prediction	(Yang et al., 2021)

#### 3.2.1 Classic blood-activating and stasis-resolving herb pairs: targeting the VEGF/VEGFR2 pathway

Representative pairs include *Boswellia carterii-Commiphora myrrha*, *Sparganium stoloniferum-Curcuma zedoaria*, and *C. zedoaria-Rheum palmatum*. Their core active components are terpenoids (boswellic acid, β-elemene), phenolic acids (emodin), and anthraquinones (emodin), with mechanisms centered on the precise inhibition of the VEGF/VEGFR2 pathway and the regulation of vascular endothelial cell proliferation (Figure 3).

**FIGURE 3:**
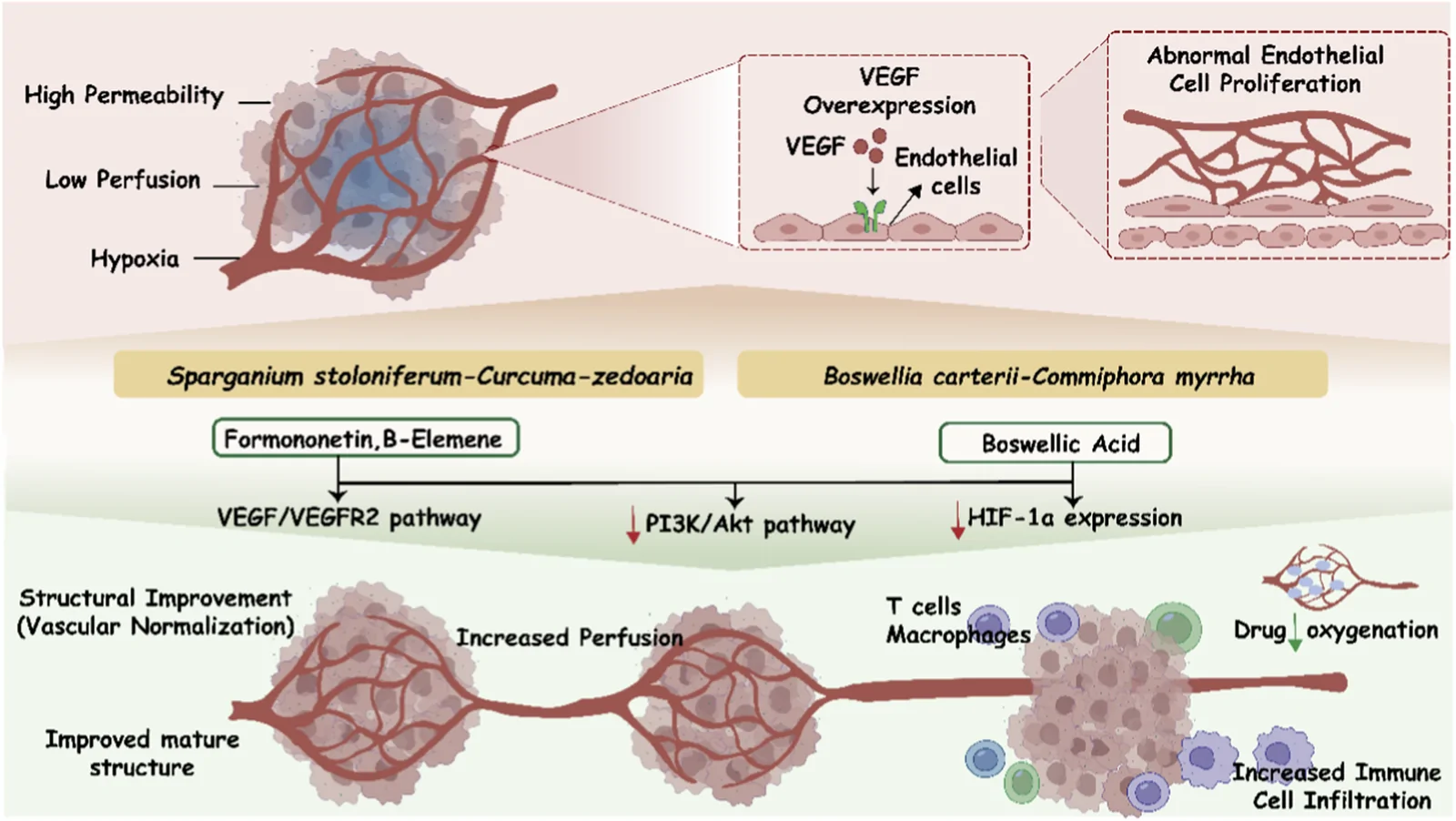
Schematic diagram of the angiogenic regulatory mechanism of blood-activating and stasis-resolving herb pairs in the TME.

##### 3.2.1.1 Boswellia carterii-Commiphora myrrha

*Boswellia carterii* Birdw. – *Commiphora myrrha* (Nees) Engl. [Burseraceae; Olibanum–Burseraceae; Myrrha] is predicted to regulate endothelial proliferation and migration via the PI3K/Akt and MAPK pathways. Core components boswellic acid and quercetin show strong binding affinity for upstream signal transducers GRB2 and YWHAE, which may interfere with growth factor signal transduction and stabilize vascular structure (Wang et al., 2020; Yao et al., 2021). **Evidence level: Level 1 (computational prediction).**

##### 3.2.1.2 Curcuma zedoaria-Rheum palmatum

*Curcuma zedoaria* (Christm.) Roscoe–*Rheum palmatum* L. [Zingiberaceae; Curcumae Rhizoma–Polygonaceae; Rhei Radix et Rhizoma] is predicted to inhibit pathological angiogenesis via the HIF-1α/VEGF axis. Core components β-elemene and obtusin downregulate HIF-1α expression, which in turn reduces tumor-derived VEGF secretion, curbs abnormal endothelial proliferation and ameliorates tumor hypoxia (Wang TT. et al., 2025). Evidence level: Level 1 (computational prediction).

##### 3.2.1.3 Sparganium stoloniferum-Curcuma zedoaria

*Sparganium stoloniferum* (Buch.-Ham. ex Graebn.) Buch.-Ham. ex Juz. – *C. zedoaria* (Christm.) Roscoe [Typhaceae; Sparganii Rhizoma–Zingiberaceae; Curcumae Rhizoma] targets PTGS2 and VEGF signaling via formononetin and β-sitosterol. PTGS2 upregulation drives tumor angiogenesis by inducing VEGF expression; inhibition of this axis is predicted to block endothelial tube formation and reduce vascular leakage (Liu et al., 2022). Evidence level: Level 1 (computational prediction).

#### 3.2.2 Mass-resolving and detoxifying herb pairs: Indirect modulation of the angiogenic microenvironment

Pairs such as *Cremastra appendiculata-Vespae Nidus*, *Fritillaria thunbergii-Bolbostemma paniculatum*, *B. paniculatum-C. appendiculata*, and *F. thunbergii-C. appendiculata* exert auxiliary regulatory effects by inhibiting the cross-talk between tumor cells and vascular endothelial cells. Their core components are saponins (tubeimoside, peimine) and phenanthrenes (colchicine).

##### 3.2.2.1 Cremastra appendiculata-Vespae Nidus

*Cremastra appendiculata* (D.Don) Makino–*Vespa mandarinia* Smith [Orchidaceae; Cremastrae Pseudobulbus–Vespidae; Vespae Nidus] inhibits MDA-MB-231 cell invasion by 34.8% via regulating the MMP-9/TIMP-1 balance (Liu et al., 2014). By restraining ECM degradation, it weakens the invasive crosstalk between tumor cells and endothelial cells, and reduces the release of pro-angiogenic factors from degraded matrix. Evidence level: Level 2 (*in vitro* validation).

##### 3.2.2.2 Fritillaria thunbergii-Bolbostemma paniculatum

*Fritillaria thunbergii* Miq. – *B. paniculatum* (Maxim.) Franquet [Liliaceae; Bulbus Fritillariae Thunbergii–Cucurbitaceae; Bolbostemmatis Rhizoma] achieves a 50.05% tumor inhibition rate in 4T1 xenografts via inhibiting the JAK1/2-STAT3 pathway (He et al., 2025). Since JAK/STAT signaling regulates macrophage polarization and inflammatory cytokine networks within the TME, its suppression indirectly reduces release of pro-angiogenic mediators such as IL-6 and TNF-α. Evidence level: Level 3–4 (*in vivo* validation + small-sample clinical observation).

##### 3.2.2.3 Bolbostemma paniculatum-Cremastra appendiculata

Plasma metabolomics in tumor-bearing mice show that this pair alters sphingolipid and glycerophospholipid metabolism, which affects cell membrane fluidity and pro-angiogenic signal transduction. Core sterol components also suppress inflammatory pathways via PTGS2, reducing pro-angiogenic factor release (Li, 2025). Evidence level: Level 2 (*in vitro* + metabolomics).

##### 3.2.2.4 Fritillaria thunbergii-Cremastra appendiculata

*Fritillaria thunbergii* Miq. – *C. appendiculata* (D.Don) Makino [Liliaceae; Bulbus Fritillariae Thunbergii–Orchidaceae; Cremastrae Pseudobulbus] is predicted to reduce pro-angiogenic mediator release via regulating steroid hormone response and IL-17 signaling (Yang et al., 2021). Evidence level: Level 1 (computational prediction).

Detailed pharmacological parameters of all included experimental studies targeting the breast cancer vascular microenvironment are summarized in Table 7.

**TABLE 7:** Detailed pharmacological parameters of experimental studies on TCM herb pairs targeting the breast cancer vascular microenvironment.

Functional category	Herb pair	Extract/Test substance	Experimental model	Cell line/Animal model	Control group setting	Concentration/Dosage range	Minimum effective Concentration/IC_50_	Intervention duration
Blood-Activating and Stasis-Resolving	*Curcuma zedoaria - Rheum palmatum*	Core active components (β-elemene, emodin, obtusin, etc.)	Computational prediction	— (network pharmacology + molecular docking)	—	—	—	—
	*Boswellia carterii - Commiphora myrrha*	Core active components (3-methoxyfuranoguaia-9-en-8-one, boswellic acid, quercetin, etc.)	Computational prediction	— (network pharmacology + molecular docking)	—	—	—	—
	*Sparganium stoloniferum - Curcuma zedoaria*	Core active components (hederagenin, formononetin, β-sitosterol, stigmasterol, trans-gondolic acid, etc.)	Computational prediction	— (network pharmacology + molecular docking)	—	—	—	—
	*Bolbostemma paniculatum - Fritillaria thunbergii*	**S2**: Fresh dichloromethane extract (sterols); **ZBA**: Total alkaloid extract (peimine, peiminine)	*In vitro* cell model and *In vivo* animal model	*In vitro*: MDA-MB-231, BT-549, MCF-7, MCF-7/ADR-RES; *In vivo*: MDA-MB-231-GFP nude mice orthotopic model	Blank control (vehicle); Positive control (Paclitaxel, Tetrandrine)	*In vitro*: **S2** 0–150 μg/mL, **ZBA** 0–150 μg/mL; *In vivo* gavage:**S2** 20, 50 mg/kg; **ZBA**15, 20 mg/kg	*In vitro*: IC_50_ not reported, significant synergy observed; *In vivo*: Low-dose combination (T20 + Z15) tumor inhibition rate 34.78%; High-dose 19.32%	*In vitro*: 48 h; *In vivo*: 8 weeks (56 days)
​	*Fritillaria thunbergii - Bolbostemma paniculatum*	Water decoction (1:1 ratio, crude drug 1 g/mL)	*In vivo* animal model	Human breast cancer 4T1 cells in BALB/c nude mice subcutaneous xenograft	Model group (0.9% NaCl gavage); Positive control (Doxorubicin 0.1 mg, i.p., once/7d)	Gavage: High 5.2 g/kg, Medium 2.6 g/kg, Low 1.3 g/kg crude drug per day	High, medium, and low dose tumor inhibition rates: **50.05%**, **28.63%**, **8.83%** (dose-dependent)	21 consecutive days
Resolving-Stasis and Detoxifying	*Cremastra appendiculata - Fritillaria thunbergii*	Core active components (β-sitosterol, stigmasterol, 2-methoxy-9,10-dihydrophenanthrene-4,5-diol, etc.)	Computational prediction	— (network pharmacology + molecular docking)	—	—	—	—
	*Cremastra appendiculata - Vespae Nidus*	Medicated serum (water decoction of crude drug 1.5 g/mL)	*In vitro* cell model	Human breast cancer MDA-MB-231 cells	Blank control (same volume saline rat serum); Positive control (Doxorubicin rat serum)	Final serum concentration **20%**	Cell killing rate **35.86%** (comparable to positive control 35.19%)	72 h
	*Bolbostemma paniculatum - Cremastra appendiculata*	Water decoction (crude drug 1 g/mL)	*In vitro* cell model and *In vivo* animal model	*In vitro*: Mouse breast cancer 4T1 cells; *In vivo*: 4T1 tumor-bearing BALB/c mice	*In vitro*: Blank control (DMEM complete medium); *In vivo*: Model group (0.9% NaCl gavage)	*In vitro*: 6.4, 3.2, 1.6, 0.8 g/L; *In vivo* gavage: Low 0.2 g/mouse, High 0.4 g/mouse	*In vitro*: 6.4 g/L and 1.6 g/L significantly inhibit cell viability (P < 0.05); *In vivo*: Significant tumor growth inhibition (metabolic changes observed)	*In vitro*: 24 h; *In vivo*: 14 consecutive days

### 3.3 Stromal microenvironment: Precision regulation from “fibrosis” to “softening”

The central contradiction of the stromal microenvironment is the activation of cancer-associated fibroblasts (CAFs) and excessive collagen deposition (Mao et al., 2021). Qi-tonifying and pathogen-eliminating herb pairs are the primary regulators of this dimension, assisted by certain blood-activating and stasis-resolving pairs that improve ECM architecture (Table 8). The *Trionyx sinensis-Curcuma zedoaria* pair has a 40-year clinical application history in breast cancer management in breast cancer treatment, but its stromal softening mechanism has only been preliminarily verified *in vitro*. Most other herb pairs in this section lack *in vivo* evidence of extracellular matrix remodeling. The long-term effects of stromal modulation on tumor progression and treatment response remain poorly understood.

**TABLE 8:** Regulatory mechanisms of TCM herb pairs on the breast cancer stromal microenvironment.

Functional categories	Herb pair	Core active components	Key targets/Pathways	Primary regulatory effect	Experimental evidence	References
Qi-Tonifying and Pathogen-Eliminating	*Trichosanthes kirilowii-Arctium lappa*	β-Sitosterol, Kaempferol	CTNNB1 (β-catenin), AKT1, TNF	Inhibits CAF activation; reduces collagen deposition	Network pharmacology + Molecular docking	(Wang and Li, 2024)
	*Fritillaria thunbergii-Trichosanthes kirilowii*	Trichosanthin, Peimine	PI3K/AKT, Bax/Bcl-2	Suppresses CAF proliferation; enhances chemosensitivity	*In vitro* + Chemotherapy synergy	(Zhang, 2023)
Blood-Activating and Stasis-Resolving	*Trionyx sinensis-Curcuma zedoaria*	Glycine, Curcumin	mTORC1/ULK1, Wnt/β-catenin	Promotes autophagy-mediated collagen degradation; softens tumor stroma	*In vitro* +40-year clinical application	(Leng et al., 2020; Feng et al., 2025; Hu et al., 2020; Hu, 2020; Yang et al., 2016)
	*Platycodon grandiflorus-Curcuma zedoaria*	Platycodin D, Curcumol	Akt/MAPK, TGF-β1/Smad7	Improves drug penetration; inhibits breast cancer metastasis	*In vitro* + Animal	(Shi et al., 2023)
	*Bupleurum chinense-Curcuma aromatica*	Saikosaponin, Curcumin	JAK/STAT3, TNF	Modulates stromal-immune crosstalk; stage-specific clinical application	Clinical formula analysis	(Li et al., 2022)

#### 3.3.1 Qi-tonifying and pathogen-eliminating herb pairs: Inhibiting CAF activation and proliferation

Represented by *Trichosanthes kirilowii-Arctium lappa* and *Fritillaria thunbergii-T. kirilowii*, these pairs inhibit CAF activation by modulating the TGF-β/Smad pathway and blocking intercellular cross-talk. Core components include flavonoids (quercetin), saponins (trichosanthin, peimine), and alkaloids (peimisine).

##### 3.3.1.1 Trichosanthes kirilowii-Arctium lappa

*Trichosanthes kirilowii* Maxim. – *A. lappa* L. [Cucurbitaceae; Trichosanthis Fructus–Asteraceae; Arctii Fructus] is predicted to inhibit CAF activation and collagen deposition via the Wnt/β-catenin pathway, mediated by β-sitosterol and kaempferol (Wang and Li, 2024). Evidence level: Level 1 (computational prediction).

##### 3.3.1.2 Fritillaria thunbergii-Trichosanthes kirilowii

Originating from the classic formula *Gualou Beimu Yin*, this pair inhibits TNBC cell proliferation, migration and apoptosis via the PI3K/AKT pathway, and synergizes with cisplatin (Zhang, 2023). Since PI3K/AKT signaling drives CAF activation, its inhibition indirectly ameliorates stromal fibrosis, reduces the physical barrier and enhances chemosensitivity. Evidence level: Level 2 (*in vitro* validation).

#### 3.3.2 Blood-activating and stasis-resolving herb pairs: improving ECM structure and drug penetration

Herb pairs like *Trionyx sinensis-Curcuma zedoaria*, *Platycodon grandiflorus-C. zedoaria*, and *Bupleurum chinense-Curcuma aromatica* reduce the ECM physical barrier by “breaking blood stasis and moving qi” or “soothing the liver and relieving depression.” Among them, the *Bupleurum-C. aromatica* pair, a representative liver-soothing and qi-regulating combination, improves the crosstalk between the stromal microenvironment and immunosuppression by modulating cytokines (Figure 4).

**FIGURE 4:**
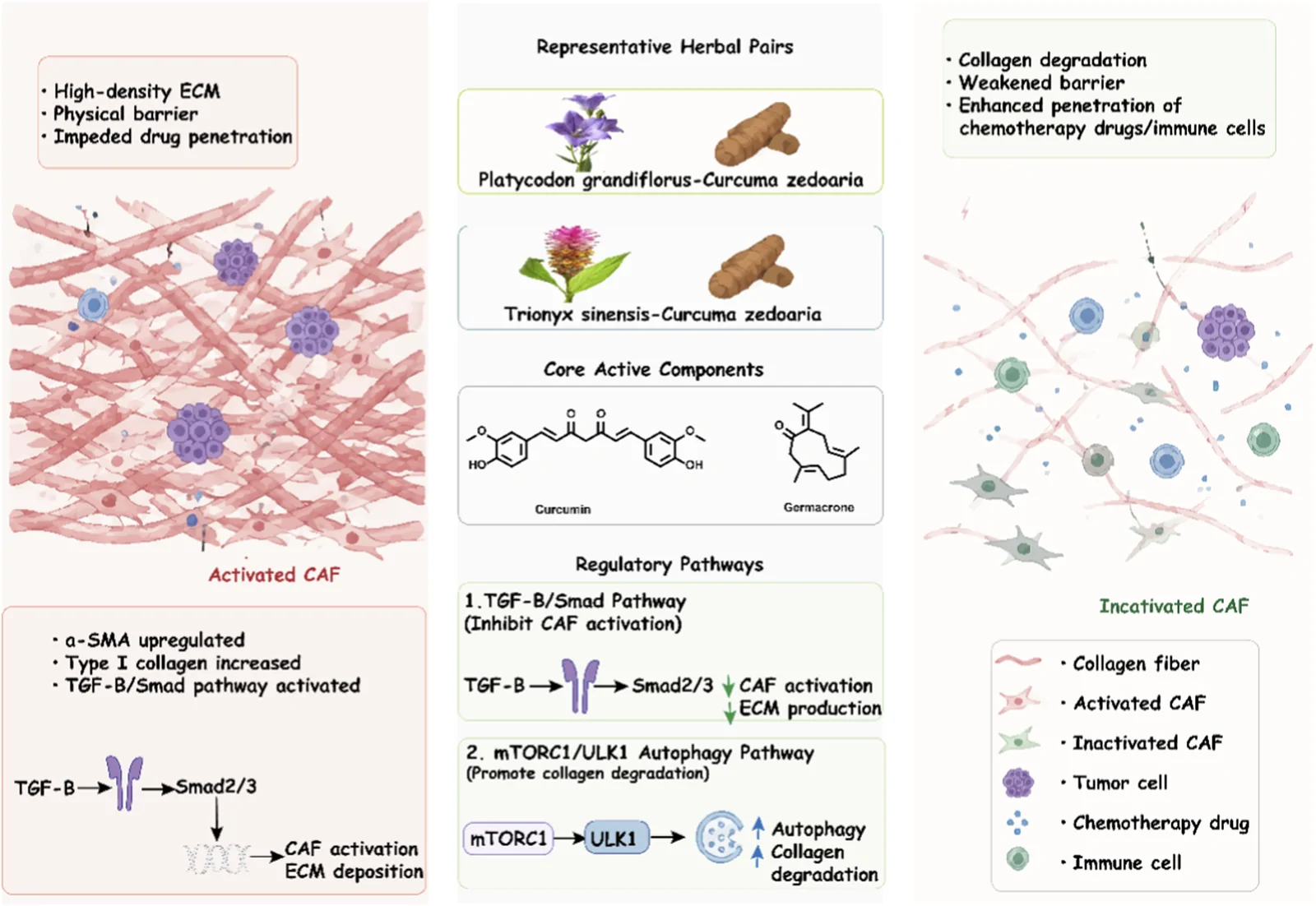
Schematic diagram of the stromal fibrosis regulatory mechanism of TCM herb pairs in the breast cancer TME.

**FIGURE 5:**
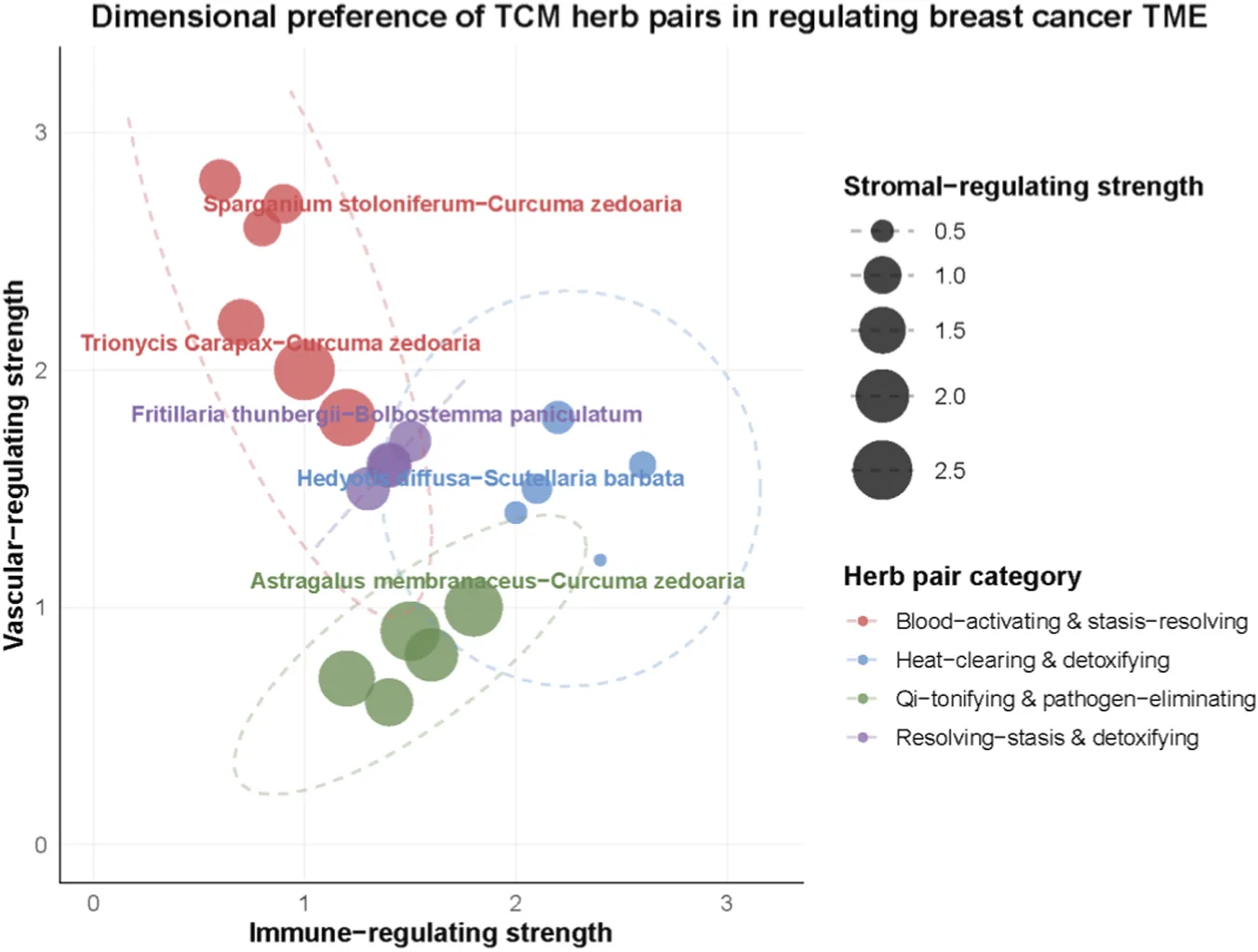
Dimensional preference clustering of 20 TCM herb pairs in regulating breast cancer TME.

##### 3.3.2.1 Trionyx sinensis-Curcuma zedoaria

*Pelodiscus sinensis* Wiegmann–*Curcuma zedoaria* (Christm.) Roscoe [Trionychidae; Trionycis Carapax–Zingiberaceae; Curcumae Rhizoma] has over 40 years of clinical application in breast cancer treatment. Its core components glycine and curcumin co-regulate the mTORC1/ULK1 pathway, promoting autophagy-mediated degradation of abnormally deposited collagen and softening the tumor stromal barrier (Feng et al., 2025; Hu et al., 2020). This stromal softening effect may improve the penetration of chemotherapeutic drugs and immune cells into tumor parenchyma. Evidence level: Level 2 + 4 (*in vitro* mechanistic validation + long-term clinical application).

##### 3.3.2.2 Platycodon grandiflorus-Curcuma zedoaria

*Platycodon grandiflorus* (Jacq.) A.DC. – *C. zedoaria* (Christm.) Roscoe [Campanulaceae; Platycodonis Radix–Zingiberaceae; Curcumae Rhizoma] modulates the TGF-β1/Smad7 pathway via platycodin D and curcumol, which inhibits epithelial-mesenchymal transition and reduces CAF-mediated ECM remodeling. When combined with nanocarriers, it further enhances tumor tissue penetration of active compounds (Shi et al., 2023). Evidence level: Level 2–3 (*in vitro* validation + *in vivo* absorption study).

##### 3.3.2.3 Bupleurum chinense-Curcuma aromatica

*Bupleurum chinense* DC. – *Curcuma aromatica* Salisb. [Apiaceae; Bupleuri Radix–Zingiberaceae; Curcumae Radix] exhibits stage-specific clinical compatibility: in the consolidation phase, it alleviates endocrine therapy-related adverse effects; in the chemoradiotherapy phase, it reduces toxicity and enhances efficacy (Li et al., 2022). By modulating cytokine networks, it improves stromal-immune crosstalk and coordinates multi-dimensional TME regulation. Evidence level: Level 4 (clinical prescription data mining).

Detailed pharmacological parameters of all included experimental studies targeting the breast cancer stromal microenvironment are presented in Table 9.

**TABLE 9:** Detailed pharmacological parameters of experimental studies on TCM herb pairs targeting the breast cancer stromal microenvironment.

Functional category	Herb pair	Extract/Test substance	Experimental model	Cell line/Animal model	Control group setting	Concentration/Dosage range	Minimum effective concentration/IC_50_	Intervention duration
Mass-Resolving and Detoxifying	*Trichosanthes kirilowii – Arctium lappa*	Core active components (arctiin, kaempferol, β-sitosterol, etc.)	Computational prediction	— (network pharmacology + molecular docking)	—	—	—	—
Liver-Soothing and Qi-Regulating	*Bupleurum chinense – Curcuma aromatica*	Clinical herbal prescription	Clinical data mining	— (network pharmacology + molecular docking)	—	—	—	—
Resolving-Stasis and Detoxifying	*Bolbostemma paniculatum – Trichosanthes kirilowii* (“Bei Mu-Gua Lou” pair)	Freeze-dried water decoction (1:1 ratio)	*In vitro* cell model	Human TNBC cell lines MDA-MB-231, MDA-MB-468	Blank control (no treatment); Positive control (Cisplatin 20 μg/mL)	Mono/Herb pair: 4, 8, 16, 32 mg/mL; Combination: Optimal IC_50_ doses	Pair (24 h) IC_50_: 8.683 mg/mL (MDA-MB-231); 11.417 mg/mL (MDA-MB-468)	24–48 h (MTT, wound healing, Transwell, Flow cytometry); 10 days (Colony formation)
Blood-Activating and Stasis-Resolving	*Trionyx sinensis-Curcuma zedoaria* (Key components: Glycine-Curcumin)	Component compatibility (Glycine, Curcumin)	*In vitro* cell model	Human TNBC cell line MDA-MB-231	Blank control (no treatment); Positive control (Everolimus, mTORC1 inhibitor)	Glycine: 120–360 mmol/L; Curcumin: 25–100 μmol/L; Combination (1/2 IC_50_ each)	Glycine IC_50_ = 262.42 mmol/L; Curcumin IC_50_ = 76.44 μmol/L	24 h (CCK-8, MDC staining, Flow cytometry, WB); 10 days (Colony formation)
Qi-Regulating and Blood-Activating	*Platycodonis Radix – Curcuma zedoaria* (Combined with Silibinin-LPNs)	Water decoction freeze-dried powder; Silibinin-loaded lipid-polymer nanoparticles (LPNs)	*In vitro* cell model and *In vivo* intestinal absorption model	Caco-2/E12 co-culture model; Mouse breast cancer 4T1 cells; BALB/c mice intestinal model	Blank control; PC single agent; NPs single agent	PC (herb pair): 0.25 + 0.5–2 + 4 mg/mL; Silibinin: 1–10 μg/mL	IC_50_ not reported; CDI <1 shows synergistic inhibitory effect on 4T1 proliferation at 7.5 and 10 μg/mL; Effective concentration for synergy is ≥7.5 μg/mL silibinin	1 h (cellular uptake); 24 h (CCK-8, wound healing); 20 min (*in vivo* intestinal perfusion)

### 3.4 Cross-dimensional synergistic effects of TCM herb pairs in modulating the TME

#### 3.4.1 Cross-dimensional action network of active components within an herb pair

Core active components within one herb pair often act on multiple TME compartments simultaneously, forming an intrinsic synergistic network (Figure 5).

Flavonoid-centered multi-target synergy: Flavonoids such as quercetin and luteolin, present across multiple herb pair categories (Du et al., 2018; Xu et al., 2020; Yan and Lyu, 2015), simultaneously inhibit the PI3K/AKT/mTOR pathway to activate anti-tumor immunity (O'Donnell et al., 2018; Nian et al., 2019), downregulate VEGF expression to curb angiogenesis (Qian et al., 2024), and suppress Wnt/β-catenin signaling to inhibit CAF activation. This “one component, multiple targets” property forms the material basis for intrinsic cross-dimensional regulation (Zhou et al., 2021).

Dual functional orientation: Pairs such as *Scutellaria barbata*–*Salvia chinensis* combine heat-clearing and blood-activating properties, coordinating immune and vascular microenvironments via a single core component.

#### 3.4.2 Inter-dimensional synergistic strategies of different herb pair categories in compound formulas

In clinical compound formulas, combining herb pairs of different functional categories according to the “sovereign-minister-assistant-envoy” principle generates multi-dimensional synergies that exceed the sum of single-category effects:

Immune activation + vascular normalization: Heat-clearing pairs activate effector immune cells, while blood-activating pairs reduce vascular leakage and improve tissue perfusion via VEGF inhibition (Hashizume et al., 2000). This combination delivers activated immune cells deeper into the tumor core, synergistically converting “cold” tumors to “hot” tumors.

Stromal softening + drug sensitization: Qi-tonifying and blood-activating pairs reduce the ECM physical barrier by inhibiting CAF activation and promoting collagen degradation, which enhances the penetration of chemotherapeutic agents and immune cells (Shi et al., 2023). Their modulation of the PTEN/PI3K/AKT pathway also produces biological synergy with chemotherapy, reversing drug resistance.

Qi-strengthening bidirectional support: Qi-tonifying pairs improve immune cell metabolic fitness to prevent exhaustion, while simultaneously inhibiting fibroblast activation to slow stromal fibrogenesis at the source (Deng et al., 2021). This holistic regulation creates a sustainable favorable microenvironment for anti-tumor therapy (Figure 6).

The multi-dimensional regulatory profiles of representative core active components underlying these synergistic effects are visualized in Figure 7, and their detailed target characteristics, involved herb pair categories and synergistic mechanisms are systematically summarized in Table 10.

**FIGURE 6:**
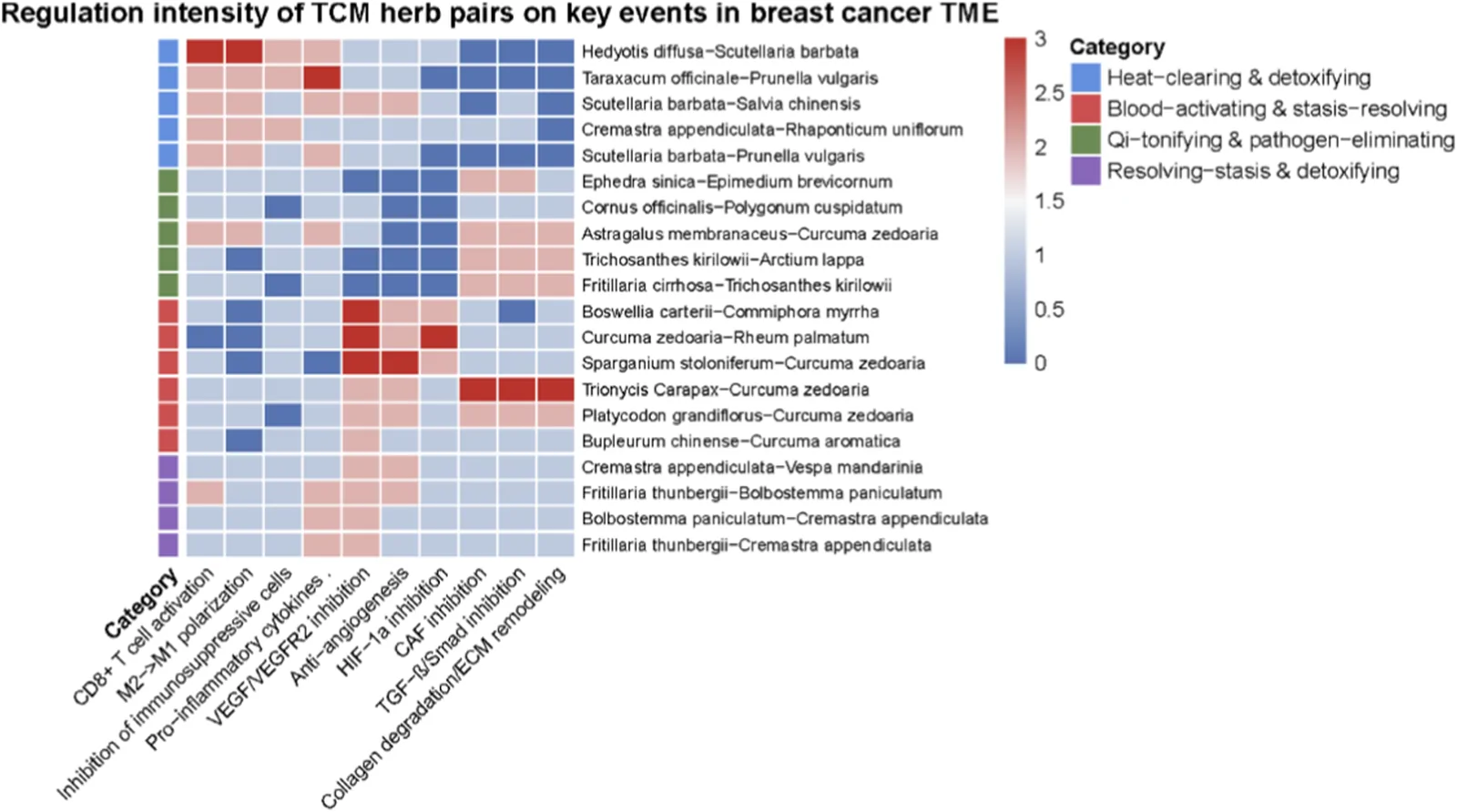
Heatmap of regulatory intensities of 20 TCM herb pairs on three dimensions of breast cancer TME.

**FIGURE 7:**
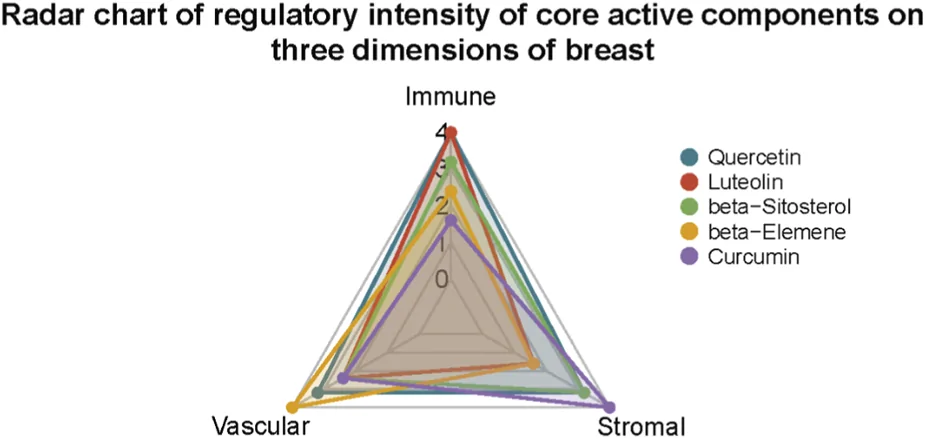
“One-stone-three-birds” effects of representative active components from TCM herb pairs on breast cancer TME.

**TABLE 10:** Multi-dimensional regulatory characteristics of core active components from TCM herb pairs in the breast cancer tumor microenvironment.

Core component	Common targets	Specific targets	Regulatory dimensions	Involved herb pair categories	Synergistic mechanism
Quercetin	PI3K/AKT, VEGF, Wnt/β-catenin	ESR1, mTOR	Immune + Vascular + Stromal	Heat-clearing, Qi-tonifying, Blood-activating	Simultaneously inhibits multiple pathways, synergistically remodeling the TME
Luteolin	PI3K/AKT, MAPK, CASP3	IL6, AKT1	Immune + Proliferation Regulation	Heat-clearing, Qi-tonifying	Enhances immune activation + induces apoptosis
β-Sitosterol	PI3K/Akt, Wnt/β-catenin	PTGS2, JUN	Immune + Stromal + Vascular	Heat-clearing, Mass-resolving	Inhibits CAF activation + anti-inflammatory + anti-angiogenesis
β-Elemene	VEGF/VEGFR2, NF-κB	STAT3	Vascular + Immune	Blood-activating	Anti-angiogenesis + modulates inflammatory factor network
Curcumin	mTORC1, ULK1, β-catenin	PI3K	Stromal + Drug Penetration	Blood-activating	Promotes collagen degradation + enhances drug delivery

#### 3.4.3 Synergistic systemic remodeling in combination with modern therapies

The combination of TCM herb pairs with contemporary anti-cancer modalities (chemotherapy, targeted therapy, immunotherapy) represents a crucial strategy to overcome the limitations of the latter and achieve systemic remodeling of the TME.

Reversing immune checkpoint inhibitor resistance: Heat-clearing and detoxifying pairs increase CD8^+^ T cell infiltration and reduce M2-type macrophages (Wang and Chan, 2025; Dong et al., 2023; Ju and Wei, 2007; Cui and Wang, 2005), priming an “immune-cold” TME for immunotherapy activity.

Enhancing chemotherapy efficacy and reducing toxicity: Blood-activating and qi-tonifying pairs promote drug delivery while alleviating chemotherapy-induced myelosuppression and gastrointestinal adverse reactions (Cheng et al., 2023).

Delaying targeted therapy resistance: The multi-target nature of herb pairs simultaneously blocks multiple compensatory signaling nodes, delaying the emergence of drug resistance.

Specific synergistic mechanisms, applicable scenarios and supporting evidence for each combination modality are systematically outlined in Table 11.

**TABLE 11:** Synergistic strategies and clinical application scenarios of TCM herb pairs combined with modern anti-cancer therapies.

Combination modality	Herb pair category	Synergistic mechanism	Clinical application scenario	Evidence support
Herb Pair + Immunotherapy	Heat-clearing and Detoxifying	Activates effector immune cells, reduces suppressive cells, and reverses TME immune inertia	Patients with no or low response to immunotherapy	*In vitro* experiments + small-sample clinical observations
Herb Pair + Chemotherapy	Qi-tonifying and Pathogen-Eliminating + Blood-Activating and Stasis-Resolving	Softens stroma to promote drug penetration, and mitigates chemotherapy toxicity (myelosuppression, GI reactions)	Neoadjuvant chemotherapy for locally advanced breast cancer/late-stage palliative chemotherapy	Animal experiments + clinical retrospective studies
Herb Pair + Targeted Therapy	Mass-Resolving and Detoxifying + Qi-tonifying and Pathogen-Eliminating	Multi-pathway synergistic inhibition to block the activation of drug-resistant bypass pathways	Drug resistance in targeted therapy for HER2-positive or HR-positive breast cancer	Network pharmacology prediction + *in vitro* validation
Herb Pair + Endocrine Therapy	Liver-Soothing and Qi-Regulating + Qi-tonifying and Pathogen-Eliminating	Modulates hormone signaling and ameliorates endocrine therapy-related adverse effects (hot flashes, insomnia)	Adjuvant endocrine therapy for hormone receptor-positive breast cancer	Clinical formula pattern analysis + small-sample observations

## 4 Nanotechnology-enabled strategies to enhance TCM herb pair efficacy

While the previous sections have highlighted the multi-component, multi-target advantages of TCM herb pairs in remodeling the TME, a critical bottleneck in translating these preclinical findings into clinical practice lies in the unfavorable pharmacokinetic profiles of their core active ingredients. To address this inherent limitation, nanotechnology offers a transformative platform (Zhang et al., 2024). This chapter delineates how nano-delivery systems can overcome the pharmacokinetic barriers of TCM active components, enhance the synergistic effects of herb pairs, and achieve targeted modulation of the breast cancer TME.

### 4.1 The pharmacokinetic bottleneck of TCM active components

As detailed in the mechanistic sections, flavonoids (e.g., quercetin, luteolin), terpenoids (e.g., curcumin, β-elemene), and sterols (e.g., β-sitosterol) are the primary mediators of TME regulation (Carullo et al., 2020). However, these compounds generally exhibit extremely poor oral bioavailability, rapid metabolism, and short plasma half-lives, severely limiting their ability to reach therapeutically effective concentrations within the tumor tissue.

Table 12 systematically summarizes the pharmacokinetic profiles of these core TME-regulating active components. From the table, it is evident that the oral bioavailability of curcumin is less than 1%, with a plasma half-life of less than 1 h; similarly, quercetin and luteolin have oral bioavailabilities below 5%. *In vitro* effective concentrations (e.g., 10–100 μM for quercetin) are frequently 50 to 1,000 times higher than the peak plasma concentrations achievable at maximum tolerated oral doses (0.1–1 μM for quercetin) (Zhang et al., 2021). Furthermore, these compounds undergo extensive phase II metabolism (glucuronidation and sulfation) in the intestine and liver, resulting in conjugates with significantly reduced or negligible biological activities. This dramatic “dose discrepancy” necessitates innovative formulation strategies to bridge the gap between *in vitro* efficacy and *in vivo* pharmacokinetics.

**TABLE 12:** Pharmacokinetic properties of core TME-regulating active components.

Active component	Oral bioavailability in humans	Peak plasma concentration (clinical dose)	*In Vitro* effective concentration	Primary metabolic pathways	Plasma half-life
Curcumin	<1%	0.01–0.1 μM (12 g/day)	5–50 μM	Glucuronidation, sulfation	<1 h
Quercetin	0.3%–5.3%	0.1–1 μM (1 g/day)	10–100 μM	Glucuronidation, methylation	1–2 h
Luteolin	<2%	0.05–0.5 μM (500 mg/day)	5–30 μM	Glucuronidation, sulfation	<2 h
Kaempferol	<3%	0.08–0.6 μM (500 mg/day)	8–40 μM	Glucuronidation, sulfation	1–3 h
β-Elemene	<5%	0.2–1 μM (400 mg/day)	10–50 μM	Hydroxylation, glucuronidation	<0.5 h

### 4.2 Nano-carrier systems for enhanced oral bioavailability and solubility

Nanotechnology provides a versatile toolbox to address the solubility and stability challenges of hydrophobic TCM components. Commonly employed nano-carriers include lipid-based nanoparticles (liposomes, solid lipid nanoparticles), polymeric nanoparticles (e.g., poly (lactic-co-glycolic acid) or PLGA), and hybrid lipid-polymer nanoparticles (LPNs). These systems offer several distinct advantages (Ferhan et al., 2022). For instance, encapsulating hydrophobic compounds such as curcumin and β-elemene within the hydrophobic core of nanocarriers significantly enhances their apparent solubility in aqueous environments. Additionally, the polymeric or lipid matrix protects the encapsulated active ingredients from premature enzymatic degradation and pH-dependent hydrolysis in the gastrointestinal tract (Zhong et al., 2020). Moreover, tailoring the degradation rate of the carrier matrix allows for sustained release of the active components, maintaining effective drug concentrations over a longer period and consequently reducing the dosing frequency.

### 4.3 Herb pair-mediated nano-delivery: a case study of the *Platycodonis grandiflorus–Curcuma zedoaria* pair

A particularly innovative strategy involves the synergistic combination of TCM herb pairs with nanomedicine, where the herb pair acts not only as a pharmacodynamic agent but also as a “promoter” or “excipient” to enhance nanoparticle absorption. The *Platycodonis grandiflorus* (Jiegeng) and *Curcuma zedoaria* (Ezhu) pair offers a representative example of this “herb pair–nanoparticle” synergistic model.

Research by Shi et al. demonstrated that co-administering the *Platycodonis grandiflorus–C. zedoaria* pair with silibinin-loaded lipid-polymer nanoparticles (LPNs) markedly enhanced oral bioavailability and therapeutic efficacy against breast cancer metastasis (Shi et al., 2023). Specifically, in a Caco-2/E12 co-culture intestinal model, the herb pair significantly promoted the internalization of fluorescently labeled nanoparticles, as confirmed by flow cytometry and confocal microscopy—an effect likely attributed to saponins in Platycodon (e.g., platycodin D) that improve nanoparticle surface properties and transiently modulate tight junctions to facilitate paracellular transport. Furthermore, *in vitro* assays using 4T1 breast cancer cells revealed that this combination exhibited a synergistic inhibitory effect on cell proliferation (CDI <1) and significantly suppressed cell migration in a time-dependent manner, with the herb pair even demonstrating a preventive effect when pre-incubated. Mechanistically, the active components of *C. zedoaria* (e.g., curcumol) and *Platycodonis grandiflorus* (e.g., platycodin D) complement nanoparticle-delivered silibinin by modulating the Akt/MAPK pathway and downregulating TGF-β1/Smad7 expression. Ultimately, this paradigm achieves a dual-action synergy by promoting absorption through the herb pair while leveraging their intrinsic anti-metastatic properties to directly inhibit tumor cells.

This paradigm illustrates that TCM herb pairs can serve as functional bio-enhancers in nano-formulations. This strategy not only improves the oral absorption of modern nanomedicines but also leverages the multi-target capabilities of the herb pair to synergistically remodel the TME and inhibit breast cancer metastasis.

### 4.4 Precision targeting to the breast cancer TME

Nanotechnology enables multiple precision targeting strategies to enhance drug accumulation specifically within the breast cancer TME. Primarily, nanocarriers with a size range of 100–200 nm can leverage the enhanced permeability and retention (EPR) effect to preferentially extravasate through the leaky, disorganized vasculature and accumulate in the interstitial space (Guo et al., 2020). To further improve tumor specificity, their surfaces can be decorated with targeting ligands, such as RGD peptides that bind to integrin αvβ3 on tumor endothelial cells, or CD44 antibodies/hyaluronic acid that target the CD44 receptor—a marker often upregulated on breast cancer stem cells and associated with tumor aggressiveness. Additionally, intelligent microenvironment-responsive nano-systems can be engineered to react to specific pathological cues, including the acidic pH (6.5–6.8) or the presence of matrix metalloproteinases (MMPs) (Landgraf et al., 2020), thereby triggering structural disassembly and the on-demand release of encapsulated TCM active components precisely at the tumor site.

### 4.5 Challenges and future perspectives

Despite the promising potential, the clinical translation of nanotechnology-assisted TCM herb pair formulations faces several challenges:

Safety and Toxicity: The long-term toxicity, biodistribution, and immunogenicity of novel nanocarrier materials (especially non-biodegradable polymers) must be rigorously evaluated before clinical application. Furthermore, while herb pairs are generally well-tolerated, the combined effect of the herb pair and nano-formulation requires comprehensive toxicological assessment.

Scalability and Quality Control: The industrial-scale production of nanomedicines poses significant manufacturing challenges, including batch-to-batch reproducibility, sterilization processes, and the stability of the encapsulated active components during long-term storage. Standardized quality control protocols need to be established.

Precision Integration with TCM Theory: Future research should focus on developing “syndrome-based precision nanomedicine.” By integrating TCM syndrome differentiation (e.g., patterns of stasis, phlegm, or toxin) with TME phenotypic profiling (e.g., CAF density, CD8^+^ T cell infiltration), nanocarrier formulations can be tailored to deliver specific active components or herb pair combinations to match the unique biological milieu of individual breast cancer patients. This “syndrome-pathology” combined approach represents the ultimate frontier for translating TCM herb pairs into precision oncology therapies.

Collectively, these nanotechnology-based strategies bridge the pharmacokinetic gap between *in vitro* bioactivity and *in vivo* therapeutic efficacy, laying a critical technical foundation for the clinical translation of TCM herb pairs in breast cancer treatment.

## 5 Discussion

This systematic review synthesizes the available evidence on the specific mechanisms and synergistic potential of TCM herb pairs from various functional categories—heat-clearing and detoxifying, blood-activating and stasis-resolving, qi-tonifying and pathogen-eliminating—in regulating the immune, vascular, and stromal microenvironments of breast cancer. The available evidence suggests that while TCM herb pairs originate from traditional experience-based compatibility, their mechanisms of action show significant alignment with modern tumor microenvironment theory, showcasing multi-dimensional and systemic regulatory advantages. Concurrently, they face critical challenges in transitioning from empirical knowledge to precision medicine.

### 5.1 Common mechanisms of herb pairs in modulating the TME

The core mechanism of TCM herb pairs in regulating the breast cancer TME follows a holistic paradigm of “multi-component synergy, multi-target interaction, cross-dimensional integration”. Aligned with TCM principles of syndrome differentiation and holistic regulation, this regulatory mode exhibits unique advantages over single-target therapies at the molecular level.

Multi-component synergy forms the material basis of efficacy. All 20 core herb pairs rely on flavonoids, terpenoids and sterols as key active constituents. Guided by the compatibility rules of “mutual reinforcement (*Xiangxu*)” and “mutual assistance (*Xiangshi*)”, these components act synergistically to bypass the limitations of single-component intervention, enabling simultaneous targeting of multiple pathological links in the TME.

Multi-target interaction is the defining mechanistic feature. Active components do not target single molecules but converge on core TME regulatory networks, with PI3K-AKT, MAPK, VEGF/VEGFR2 and TGF-β/Smad as shared convergent nodes (Wang XY. et al., 2025; Zhang XG. et al., 2023). Through these pathways, herb pairs of different functional categories coordinately regulate cellular and non-cellular TME components, correcting aberrant homeostasis and overcoming the drug resistance bottleneck of single-target therapies.

Cross-dimensional integration embodies the holistic nature of TCM regulation. Despite distinct compartmental preferences, most herb pairs exert cross-dimensional synergy. This action pattern matches the intertwined pathological features of the breast cancer TME, providing modern biological evidence for the TCM holistic regulation theory.

Furthermore, the multi-component, multi-target profile of herb pairs enables systematic coping with TME heterogeneity. For example, quercetin simultaneously targets multiple signaling pathways (Li et al., 2024; Maurya and Vinayak, 2015), while β-elemene modulates a spectrum of inflammatory factors to holistically remodel the immune microenvironment (Feng et al., 2024).

### 5.2 Critical analysis of evidence levels and methodological limitations

A fundamental limitation of current TCM herb pair research is the overall low methodological quality of primary studies, which severely undermines the reproducibility and reliability of reported findings. Our systematic quality assessment of 78 included studies reveals pervasive weaknesses across all four evaluated dimensions (Table 3), spanning botanical authentication, constituent quantification, analytical technical level and extraction method disclosure.

The most critical gap lies in the poor quality of constituent reporting. Only 29.5% of included studies identified constituents via experimental mass spectrometry (UPLC-MS/MS, Q-TOF-MS or equivalent), while 70.5% derived their active constituent lists exclusively from public database predictions in network pharmacology studies, with no experimental validation. This overreliance on computational predictions creates three interconnected layers of bias that fundamentally distort mechanistic conclusions (Diao et al., 2026). First, database structural bias disproportionately overrepresents chemically stable, well-annotated flavonoids such as quercetin and luteolin while underrepresenting less studied pharmacologically relevant compounds. Second, widespread pan-assay interference substance (PAINS) issues produce frequent false-positive signals in *in vitro* validation assays, via non-specific binding, protein aggregation or fluorescence quenching. Third, a self-reinforcing feedback loop propagates false-positive predictions across interconnected databases, leading to cumulative amplification of bias over time. It must be emphasized that network pharmacology and molecular docking are exclusively hypothesis-generating tools; all conclusions based solely on these methods are tentative and require rigorous experimental verification.

Compounding the constituent identification problem is the widespread lack of botanical standardization. Only 15.4% of studies explicitly reported both the full Latin binomial with author citation and the exact medicinal part used. Different medicinal parts of the same plant species can have drastically different chemical profiles and pharmacological activities; this widespread lack of standardization introduces enormous inter-study heterogeneity and makes direct comparison and replication of results nearly impossible.

To objectively evaluate the strength of the evidence presented in this review and address the issue of equal weighting of different evidence types, we established a four-level evidence grading system based on evidence-based medicine principles:

Level 1 (Lowest evidence): Network pharmacology prediction and molecular docking
Level 2: *In vitro* cell culture experiments
Level 3: *In vivo* animal model experiments
Level 4 (Highest evidence): Human clinical studies (small-sample observations, clinical trials)

Based on this system, we systematically assessed the evidence strength of all 20 core herb pairs, and the distribution is summarized in Table 13.

**TABLE 13:** Evidence level distribution of 20 core anti-breast cancer herb pairs.

Evidence level	Number of herb pairs	Percentage	Representative herb pairs
Level 1	6	30%	*Ephedra sinica-Epimedium brevicornu*, *Cornus officinalis-Polygonum cuspidatum*, *Curcuma zedoaria-Rheum palmatum*
Level 2	5	25%	*Scutellaria barbata-Salvia chinensis*, *Cremastra appendiculata-Rhaponticum uniflorum*, *Cremastra appendiculata-Vespae Nidus*
Level 3	6	30%	*Hedyotis diffusa-Scutellaria barbata*, *Taraxacum mongolicum-Prunella vulgaris*, *Sparganium stoloniferum-Curcuma zedoaria*, *Bupleurum chinense-Curcuma aromatica*
Level 4	3	15%	*Trionyx sinensis-Curcuma zedoaria*, *Fritillaria thunbergii-Bolbostemma paniculatum*

Our critical analysis revealed significant heterogeneity in the evidence quality of current research, with distinct limitations associated with each evidence level:

Limitations of computational predictions (Level 1): As discussed above, Level 1 findings have high false-positive rates and cannot reflect the complex pharmacokinetic interactions and synergistic effects of multiple components *in vivo*. No mechanistic conclusions should be drawn solely from Level 1 evidence.

Translation gap of preclinical studies (Levels 2–3): Most preclinical findings are based on immortalized cell lines and immunodeficient xenograft models, which cannot fully recapitulate the heterogeneity and immune microenvironment of human breast cancer. Approximately 90% of promising preclinical cancer drugs fail in clinical trials, highlighting the need for extreme caution when extrapolating these results to human patients.

Methodological limitations of clinical studies (Level 4): The existing clinical evidence is limited to small-sample observational studies and case series, lacking large-scale, multicenter, randomized controlled trials (RCTs) with standardized intervention protocols and objective efficacy endpoints. Most studies do not report long-term survival outcomes or adverse event data.

Our four-level evidence grading system further quantifies these limitations. The analysis shows that 30% of the 20 core herb pairs are supported only by Level 1 computational predictions, and only 15% have supporting Level 4 clinical evidence. The vast majority of mechanistic claims remain at the preclinical stage with high translational uncertainty. Collectively, these methodological limitations mean that the mechanistic findings reviewed herein should be interpreted as indicative trends rather than established facts, and must be validated by rigorously designed, standardized preclinical and clinical studies.

### 5.3 Pharmacokinetic limitations of core active components

Even when preclinical bioactivity is confirmed, clinical translation of TCM herb pairs is severely hampered by the unfavorable pharmacokinetic properties of their core active constituents. Flavonoids and terpenoids, the primary mediators of TME regulation identified in this review, all exhibit extremely low oral bioavailability. As quantitatively summarized in Table 12, *in vitro* effective concentrations are 50–1,000 times higher than peak plasma concentrations achievable at maximum tolerated oral doses in humans. This dramatic dose mismatch represents the single largest translational barrier, meaning most TME regulatory effects observed in cell culture cannot be replicated at clinically relevant doses.

Absorbed parent compounds also undergo rapid phase II metabolism in the liver and intestine to form glucuronide and sulfate conjugates with markedly reduced biological activity, further weakening *in vivo* efficacy. This dual limitation of low exposure and metabolic inactivation explains why many promising preclinical findings fail to translate into clinical benefits. Nanotechnology-enabled delivery strategies, which reshape pharmacokinetic profiles and achieve targeted TME delivery via biocompatible nanocarriers, represent a transformative solution to this fundamental challenge.

### 5.4 Clinical translation challenges: standardization, safety and evidence gaps

Beyond preclinical and pharmacokinetic limitations, several critical clinical issues must be addressed before TCM herb pairs can be widely adopted as evidence-based therapies for breast cancer treatment. These challenges, which have been largely overlooked in previous reviews, represent the final and most formidable barrier to clinical translation.

#### 5.4.1 Lack of high-quality clinical evidence

The foremost barrier is the absence of large-scale, multicenter randomized controlled trials demonstrating survival benefits. All available clinical data derive from small, single-center observational studies with inherent selection bias, and most report only surrogate endpoints (quality of life improvement, chemotherapy toxicity alleviation, immune parameter modulation) rather than hard clinical endpoints such as overall survival or progression-free survival. Furthermore, efficacy data stratified by breast cancer molecular subtype and TME phenotype remain scarce, impeding the development of precision treatment strategies.

#### 5.4.2 Standardization and quality control of herbal preparations

Herbal preparation quality varies widely across the full production chain: differences in cultivation conditions, harvesting time, traditional processing and potential contamination can cause up to 10-fold variation in active constituent content. No internationally unified quality control standard for TCM herb pairs has been established; this variability not only invalidates cross-study comparisons but also carries latent safety risks for patients.

#### 5.4.3 Safety and drug-drug interactions

The long-term safety of most herb pairs has not been systematically evaluated in large clinical cohorts, with some constituents associated with hepatotoxicity, nephrotoxicity and allergic reactions. Most critically, many active components modulate cytochrome P450 enzymes and ABC transporters, which may alter the metabolism and intracellular exposure of concurrent chemotherapeutic or endocrine agents, resulting in either attenuated efficacy or amplified toxicity. Systematic drug interaction studies are therefore a prerequisite for combined clinical application.

#### 5.4.4 Lack of predictive biomarkers

No validated biomarkers currently exist to identify patient populations most likely to benefit from TCM herb pair treatment, leading to empirical, non-personalized clinical prescribing. Future work should develop an integrated “syndrome-pathology” prediction model combining TME phenotypic markers (PD-L1, CAF density, microvessel density) and TCM syndrome differentiation, to enable precise patient-herb matching.

### 5.5 Overall limitations of current research

Beyond evidence quality, pharmacokinetic and clinical translation barriers, three fundamental limitations persist at the preclinical level, rooted in the complexity of herbal formulations and the inherent heterogeneity of the breast cancer tumor microenvironment (TME).

First, component-target associations remain ambiguous and synergistic mechanisms are poorly defined. Network pharmacology has predicted hundreds of putative active components and thousands of potential targets for the 20 core herb pairs, but the vast majority lack functional validation. Most studies focus on a small set of well-characterized compounds (quercetin, luteolin, curcumin) while neglecting less abundant but pharmacologically critical constituents. The molecular basis of synergistic interactions between components remains unclear, and optimal compatibility ratios are still based on empirical tradition rather than quantitative pharmacodynamic/pharmacokinetic evidence.

Second, TME heterogeneity is insufficiently addressed. Almost all preclinical studies rely on a limited number of immortalized cell lines (predominantly MDA-MB-231 and MCF-7) and homogeneous immunodeficient xenograft models, which fail to recapitulate the diverse structural and immunological phenotypes of human breast cancer. The differential efficacy of herb pairs across distinct TME subtypes (“immune-hot”, “immune-excluded”, “immune-cold”) remains largely unexplored, and this one-size-fits-all paradigm impedes precision clinical application.

Third, preclinical research models have inherent limitations. Most mechanistic studies use traditional 2D cell cultures that lack native 3D architecture, cell-cell crosstalk and extracellular matrix components. Most *in vivo* studies employ immunodeficient mice, which cannot fully evaluate the immunomodulatory effects that represent a core mechanism of TCM herb pairs. More clinically relevant models such as patient-derived xenografts and tumor organoids are rarely applied in this field.

### 5.6 Future research directions

To address the comprehensive limitations outlined above and advance TCM herb pair research from empirical observation to evidence-based precision medicine, future investigations should focus on three interconnected priority areas:

Precision Validation of Basic Mechanisms: Prioritize rigorous *in vitro* and *in vivo* validation for the 6 herb pairs currently supported only by Level 1 network pharmacology evidence, with specific measures to eliminate false-positive results caused by PAINS compounds and database structural bias. Employ CRISPR-Cas9 gene editing, quantitative proteomics, and the standardized Chou-Talalay method to establish causal component-target-effect relationships and optimize herb pair compatibility ratios based on pharmacodynamic principles rather than traditional empirical experience.

Construction of Translational Technology Systems: Establish a panel of clinically relevant preclinical models representing the full spectrum of breast cancer TME phenotypes (“immune-hot”, “immune-excluded”, “immune-cold”), and widely adopt patient-derived organoids and PDX models to overcome the limitations of traditional 2D cell culture and immunodeficient mouse models. Accelerate the development of stimuli-responsive nanocarriers to improve the oral bioavailability and tumor targeting of poorly absorbable active components (curcumin, quercetin, etc.), and conduct comprehensive long-term safety evaluations for candidate nano-formulations.

Advancement of Clinical Translation Infrastructure: Develop internationally recognized standardized extraction, processing, and quality control protocols for TCM herb pairs, including strict testing for contaminants and adulterants. Initiate well-designed, multicenter randomized controlled trials using overall survival and progression-free survival as primary endpoints, with pre-specified subgroup analyses based on breast cancer molecular subtypes and TME phenotypes. Finally, integrate traditional TCM syndrome differentiation with modern molecular profiling to develop “syndrome-pathology” combined predictive biomarkers for precision patient-herb matching.

## 6 Conclusion

TCM herb pairs of different therapeutic properties demonstrate clear dominant functional preferences in regulating the three dimensions of the breast cancer TME. Heat-clearing and detoxifying herb pairs chiefly reshape the immune microenvironment by activating effector immune cells and inhibiting immunosuppressive cells. Blood-activating and stasis-resolving herb pairs concentrate on core pathways like VEGF/VEGFR2 to restore the vascular microenvironment. Qi-tonifying and pathogen-eliminating herb pairs predominantly regulate the stromal microenvironment by inhibiting CAF activation and reducing collagen deposition. Simultaneously, all categories exhibit cross-dimensional synergistic effects, conferring an advantage of multi-dimensional integrative regulation.

With their unique operational model of “multi-component synergy–multi-target interaction–cross-dimensional integration”, TCM herb pairs demonstrate significant potential in systematically remodeling the breast cancer TME and promoting integrative Chinese-Western medicine treatment. They can specifically ameliorate multiple pathological abnormalities of the TME and synergize with modern therapies to achieve therapeutic goals of enhancing efficacy, reducing toxicity, and reversing drug resistance.

However, it must be emphasized that most of the evidence presented in this review comes from preclinical studies, and the clinical efficacy and safety of these herb pairs remain to be confirmed in large-scale, multicenter, randomized controlled trials. Future research should prioritize the clinical translation of herb pairs with strong preclinical evidence, establish standardized quality control systems and efficacy evaluation criteria, and develop nano-targeted delivery systems to improve the bioavailability and tumor targeting of TCM active components.

A clinical translation pathway based on “syndrome differentiation-guided herb pair selection–precision TME modulation–synergistic efficacy enhancement” is proposed, in which nano-targeted delivery systems act as a core enabling technology to resolve the pharmacokinetic gap between preclinical efficacy and clinical application. This entails guiding the selection of herb pairs by TCM syndromes integrated with TME phenotypic profiling to achieve precise “syndrome + pathology” based pair selection. Ultimately, constructing synergistic therapeutic regimens that combine herb pairs with modern therapies will facilitate effective translation from foundational research to clinical application, propelling the integrative treatment of breast cancer toward a more precise and systematic paradigm.
